# Three-dimensional morphologic and molecular atlases of nasal vasculature

**DOI:** 10.1038/s44161-023-00257-3

**Published:** 2023-03-20

**Authors:** Seon Pyo Hong, Myung Jin Yang, Jung Hyun Bae, Du Ri Choi, Young-Chan Kim, Myeon-Sik Yang, Byungkwan Oh, Kyung Won Kang, Sang-Myeong Lee, Bumseok Kim, Yong-Dae Kim, Ji Hoon Ahn, Gou Young Koh

**Affiliations:** 1 Center for Vascular Research, Institute for Basic Science, Daejeon, Republic of Korea; 2 Graduate School of Medical Science and Engineering, Korea Advanced Institute of Science and Technology (KAIST), Daejeon, Republic of Korea; 3 Laboratory of Veterinary Pathology, College of Veterinary Medicine, Jeonbuk National University, Iksan, Republic of Korea; 4 Division of Biotechnology, College of Environmental and Bioresources, Jeonbuk National University, Iksan, Republic of Korea; 5 Laboratory of Veterinary Virology, College of Veterinary Medicine, Chungbuk National University, Cheongju, Republic of Korea; 6 Department of Otorhinolaryngology-Head and Neck Surgery, College of Medicine, Yeungnam University, Daegu, Republic of Korea; 7 Regional Center for Respiratory Diseases, Yeungnam University Medical Center, Daegu, Republic of Korea

**Keywords:** Cardiovascular biology, Molecular medicine

## Abstract

Understanding the function of the nasal vasculature in homeostasis and pathogenesis of common nasal diseases is important. Here we describe an extensive network of venous sinusoids (VSs) in mouse and human nasal mucosa. The endothelium of the VSs expressed Prox1 (considered to be a constitutive marker of lymphatic endothelium) and high levels of VCAM-1 and exhibited unusual cell-to-cell junctions. VSs are supported by circular smooth muscle cells (SMCs) and surrounded by immune cells. The nasal mucosa also showed a rich supply of lymphatic vessels with distinctive features, such as the absence of the lymphatic marker LYVE1 and sharp-ended capillaries. In mouse models of allergic rhinitis or acute Coronavirus Disease 2019 (COVID-19) infection, Prox1^+^ VSs were regressed or compromised. However, in aged mice, the VSs lost the SMC support and were expanded and enlarged. Our findings demonstrate three-dimensional morphological and molecular heterogeneities of the nasal vasculature and offer insights into their associations with nasal inflammation, infection and aging.

## Abstract (web summary)

Hong et al. provide three-dimensional morphologic and molecular atlases of the mouse and human nasal vasculature by combining immunofluorescence staining of whole-mounted nasal mucosal tissue with single-cell RNA sequencing analysis of isolated endothelial cells. The data show a complex network of venous sinusoids, embryologically derived from blood vessels but lined by endothelial cells expressing the lymphatic marker Prox1, which were regressed or compromised in mouse models of rhinitis and COVID-19 infection.

## Main

The nose is an intricate organ with multifaceted functions, including olfaction, clearance of pathogens and chemicals and passage, humidification, warming and filtration of air. To execute these fundamental functions, the nose has a well-equipped epithelium and unique vascular and neural control systems^1–4^. The nasal vascular system consists of resistance and capacitance vessels distributed mainly in the respiratory mucosa and dynamically regulated by the autonomic nervous system^1–4^. Small arteries, arterioles and arteriovenous anastomoses serve as the resistance vessels for regulating blood flow, and venous sinusoids (VSs) serve as the capacitance vessels for reserving blood before venous return^1–4^. The extensive network of VSs is the primary regulator of nasal airflow resistance in the nasal cycle^5,6^, which is the spontaneous congestion and decongestion of the nasal mucosa after the dilation and constriction of VSs during the day. This cycle is present in 70–80% of healthy adults, with congestion of one side accompanied by reciprocal decongestion of the contralateral side of the nose^5^.

The respiratory mucosa represents the largest area of the nasal cavity. It serves as a frontline barrier to inhaled exogenous dust and pathogens, vulnerabilities and insults that cause substantial damage to the epithelium and shorten its lifetime^1–3,7^. Although basal stem cells give rise to progenitor cells that rapidly proliferate and differentiate into mature epithelial cells, the barrier function can be weakened by frequent nasal diseases, such as viral infection and allergic rhinitis (AR), which allow pathogen entry into the submucosa and induce immune reactions. Maintaining the adequate function of air conditioning, barrier, stemness and immunity necessitates a sufficient and suitable vascular system. Confirming the importance of the nasal vascular system, advances in the understanding of nasal vasculature have been made progressively for the last 150 years. In 1885, Zuckerkandl made hand-drawn three-dimensional (3D) color renderings of the nasal blood vessels in humans and sheep^8^, providing details about this vasculature’s morphological characteristics, patterns and networks. Moreover, the connections and anastomoses from arteries to veins in the nasal mucosa have been delineated using vascular corrosion casting in several species of large animals^9,10^. The polarized distribution of fenestrations in the capillary endothelial cells (ECs) and the diverse alignments of muscle fibers in the vascular wall of the nasal mucosa also have been defined^11^. Despite the clarifications of the gross morphological heterogeneity in the nasal blood vessels, their spatial distribution and interconnection, cellular features, molecular heterogeneity at single-cell level, genesis and development and alteration in response to infection or inflammation have been inadequate or poorly understood.

Lymphatic vessels (LVs) serve as conduits for the drainage of fluid and immune cells from surrounding peripheral tissues to the central regulatory system. Recent discoveries of novel functions for LVs have greatly changed perspectives on the lymphatic vasculature^12–14^. LVs show remarkable plasticity and heterogeneity, reflecting their functional specialization in controlling the tissue microenvironment. LVs are abundantly distributed in the mucosa of the respiratory tract, including the trachea and lungs^15–18^. These LVs also show prominent plasticity and heterogeneity in response to pathological conditions, such as injury, inflammation and severe fibrosis^15–18^. We and others recently found that multi-ciliated cells of the nasal respiratory epithelium are the primary targets of severe acute respiratory syndrome coronavirus 2 (SARS-CoV-2) infection in the early stage of Coronavirus Disease 2019 (COVID-19)^19,20^. Several nasal spray vaccines against SARS-CoV-2 are in active development^21^, but it is unknown how the vaccines or shed virus from infected cells drain into the LVs of the nasal cavity and reach draining lymph nodes (LNs) and nasopharyngeal-associated lymphoid tissue (NALT). Although several studies described the distribution of LVs and proposed their roles in the nasal cavity^1,3,22^, their distribution at high spatial resolution, specific cellular features, molecular heterogeneity at single-cell level, immune surveillance role and alteration to chronic inflammation are unknown.

In this study, we aimed to define the morphological and molecular heterogeneities of the nasal vasculature by immunofluorescence staining (IFS) of whole-mounted nasal mucosal tissue combined with single-cell RNA sequencing (scRNA-seq) analysis of isolated ECs derived from mouse nasal mucosa. We also compared the vascular heterogeneity in mouse to that in the human nasal mucosa. Because VSs constitute a major fraction of vascular volume in the nasal mucosa, we focused on determining how they are distributed and connected with other vessels and interact with different cell types, when they emerge during embryonic development, and how they are differentially altered in an AR model, in a SARS-CoV-2 infection model or in aging.

## Results

### 3D morphological atlas of Prox1^+^ VSs in the mouse nasal mucosa

**Fig. 1:**
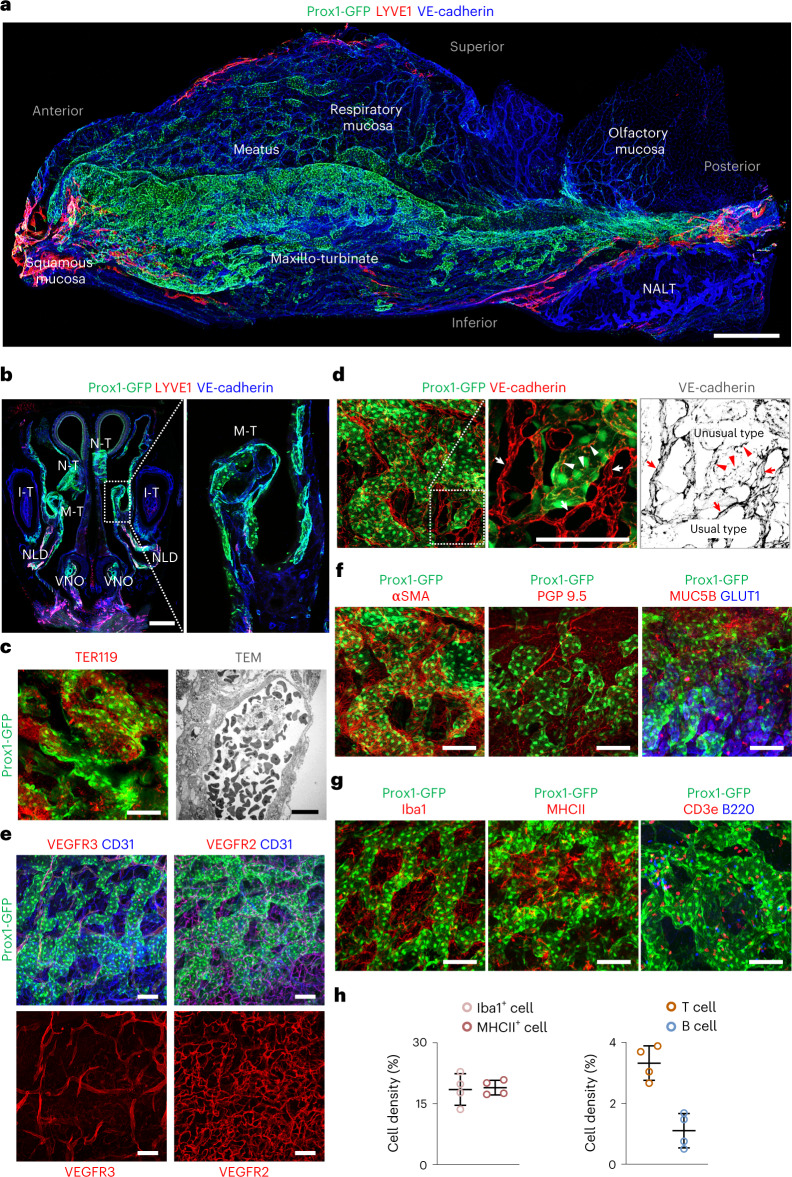
3D morphology of Prox1^+^ VSs in the mouse nasal cavity. **a**,**b**, Images showing distributions of Prox1^+^ VSs and LYVE1^+^ LVs at the indicated areas and regions of whole-mounted lateral side (**a**) and cross-sectioned (**b**) nasal cavity of Prox1-GFP reporter mice. The area in the dash-lined box is magnified and shown as the right panel. N-T, naso-turbinate; M-T, maxillo-turbinate; I-T, incisor teeth; NLD, nasolacrimal duct; VNO, vomeronasal organ. Scale bars, 500 μm. **c**, Image showing Prox1^+^ VSs filled with TER119^+^ RBCs (left). Scale bars, 100 μm. Image showing transmission electron micrograph (TEM) of VS filled with blood cells. Scale bars, 10 μm. **d**–**g**, Images showing distributions of unusual (irregularly distributed VE-cadherin) intercellular junctions (white and red arrowheads) in Prox1^+^ VSs, usual (regularly distributed VE-cadherin) intercellular junctions (white and red arrows) in surrounding capillaries and αSMA^+^ circular SMCs, PGP9.5^+^ peripheral nerve fibers, MUC5B^+^/GLUT1^+^ mucosal glands, Iba1^+^ macrophages, MHCII^+^ monocytic phagocytes, CD3e^+^ T cells, B220^+^ B cells and VEGFR3 and VEGFR2 in or around Prox1^+^ VSs in the whole-mounted nasal cavity of Prox1-GFP reporter mice. Scale bars, 100 μm. **a**–**g**, Similar findings were obtained from *n* = 7–10 mice per group from 3–5 independent experiments. **h**, Comparisons of relative cell density of Iba1^+^ cells, MHCII^+^ cells, CD3e^+^ T cells and B220^+^ B cells in the nasal mucosa. Each dot indicates a value from one mouse and *n* = 4 mice from two independent experiments. Bars indicate mean ± s.d. Source data

**Extended Data Fig. 1:**
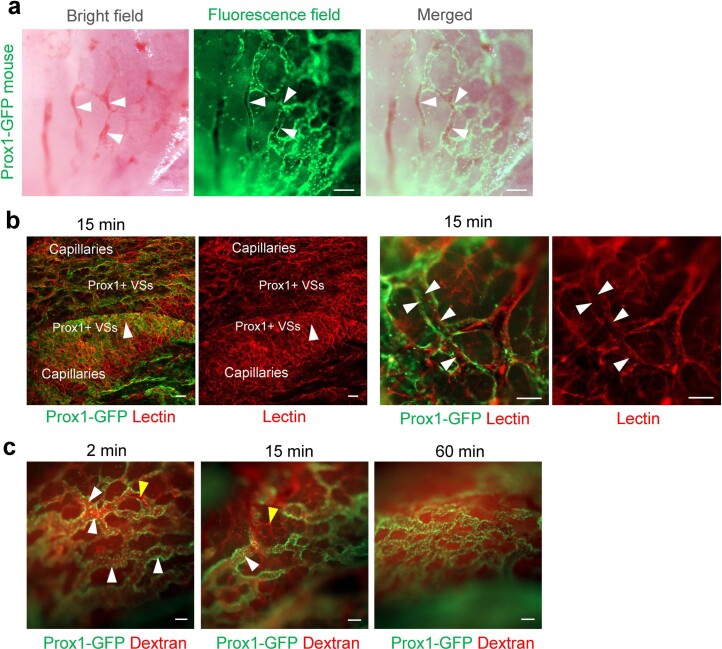
Prox1^+^ VSs have rapid and plentiful blood flow in the mouse nasal mucosa. **a**, Image showing Prox1^+^ VSs bearing blood (white arrowheads). Scale bars, 100 μm. **b**, Images showing Tomato-lectin binding in the Prox1^+^ VSs (white arrowheads) and capillaries at 15 min after i.v. injection. Scale bars, 100 μm. **c**, Images showing 2,000 kDa dextran in the Prox1^+^ VSs (white arrowheads) and capillaries (yellow arrowheads) at indicated time points after i.v. injection. Scale bars, 100 μm. Note that dextran is highly seen at 2 min, reduced at 15 min, and rarely seen at 60 min inside the Prox1^+^ VSs after the injection, while it is highly seen at 2 and 15 min in the capillaries. **a**–**c**, Similar findings were obtained from n = 4–5 mice/group from two independent experiments.

We initially attempted to determine the distribution of LVs in the nasal cavity using prospero-related homeobox 1 (Prox1)-green fluorescent protein (GFP) reporter mice, because this system allows for precise and accurate LV tracing in most organs^23,24^. However, IFS analysis of whole-mounted and cross-sectioned nasal cavity revealed that most Prox1^+^ vessels were unexpectedly large-diameter (∼50–150-μm) VSs, negative for lymphatic vessel endothelial hyaluronan receptor 1 (LYVE1) and positive for VE-cadherin, extensively distributed in most of the nasal mucosa and particularly enriched in the nasal and maxillary turbinates (Fig. 1a,b). Both IFS and transmission electron microscopy analyses showed red blood cells (RBCs) filling the lumen of these vessels (Fig. 1c), indicating blood flow and not lymph flow (that is, not LVs). Indeed, direct fluorescence stereomicroscopic imaging and perfusion analyses with lectin and dextran revealed that the VSs had a relatively high and rapid blood flow similar to the surrounding capillaries (Extended Data Fig. 1). Based on these features, we designated these Prox1^+^ vessels as ‘Prox1^+^ VSs’. In comparison, Prox1^+^/ LYVE1^+^/VE-cadherin^+^ LVs were distributed mainly in the squamous mucosa located in the anterior portion of the nasal cavity and the surrounding area of NALT (Fig. 1a).

**Extended Data Fig. 2:**
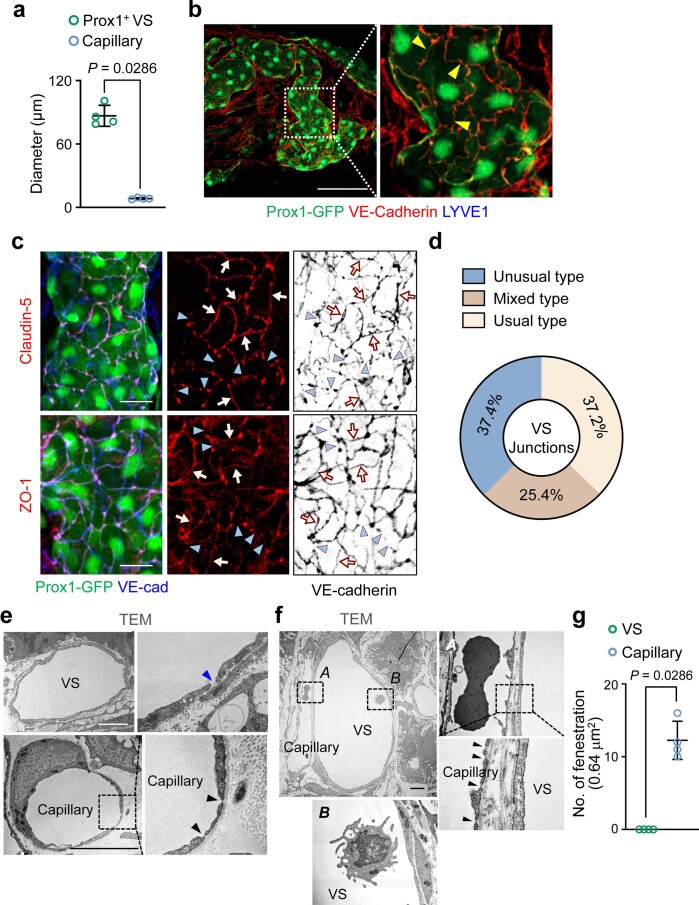
Features of diameter, EC junction, shape and fenestration in the mouse nasal vasculature. **a**, Comparison of diameter between Prox1^+^ VS and capillary in the mouse nasal mucosa. Each dot indicates a value from one mouse and n = 4 mice from two independent experiments. Data are mean ± SD. *P* values versus Prox1^+^ VS by two-tailed Mann-Whitney *U* test. **b**, Images showing irregular distribution of VE-cadherin (yellow arrowheads) in the ECs of Prox1^+^ VSs. Scale bars, 100 μm. Two independent experiments in n = 4 mice show similar findings. **c**, Representative images showing distributions of unusual (sky blue arrowheads) and usual (beige arrows) intercellular junctions in the Prox1^+^ VSs distinguished by distribution patterns of VE-cadherin, claudin-5 and ZO-1. Note co-localization of VE-cadherin and claudin-5 or ZO-1 and atypical circle-shaped ECs. Scale bars, 20 μm. Two independent experiments in n = 4 mice show similar findings. **d**, Comparison of EC intercellular junction types based on VE-cadherin distribution in the Prox1^+^ VSs. n = 4 mice from two independent experiments. **e**, **f**, TEM images showing open EC junction (blue arrowheads) in VS and fenestrations (black arrowheads) in capillary of the mouse nasal mucosa. Each area of the dash-lined box is magnified and shown as the right panel. Scale bars, 4 μm. Two independent experiments in n = 4 mice show similar findings. **g**, Comparison of number of fenestration between VS and capillary in the mouse nasal mucosa. Each dot indicates a value from one mouse and n = 4 mice from two independent experiments. Bars indicate mean ± SD. *P* values versus Prox1^+^ VS by two-tailed Mann-Whitney *U* test. Source data

**Extended Data Fig. 3:**
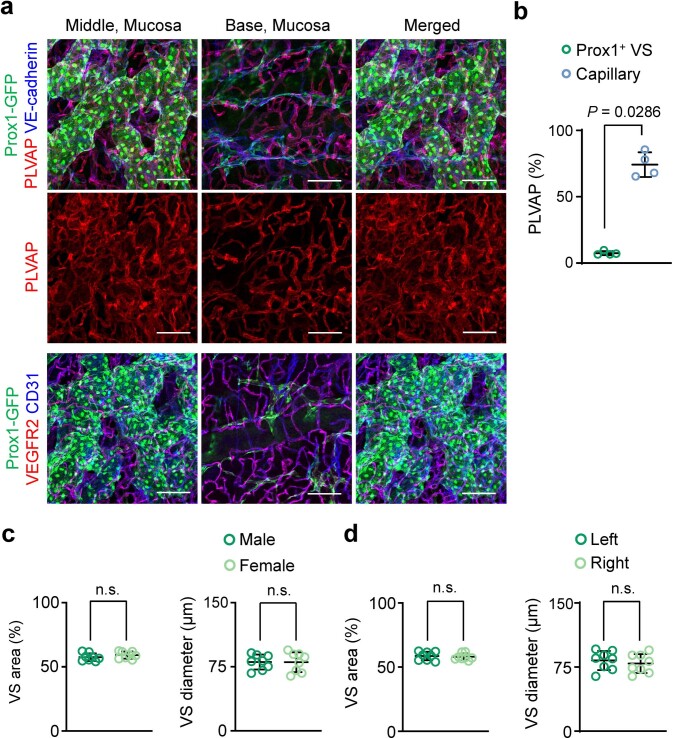
Distinct markers for the mouse nasal vasculature and the features of Prox1^+^ VS are not different by sex and the side. **a**, Images showing PLAVP^−^/VEGFR2^−^/Prox1^+^ VS distribution at the middle portion, while PLAVP^+^/VEGFR2^+^ capillary at the base portion of the mouse nasal mucosa. Scale bars, 100 μm. Two independent experiments in n = 4 mice show similar findings. **b**, Comparison of PLVAP level between Prox1^+^ VS and capillary in the mouse nasal mucosa. Each dot indicates a value from one mouse and n = 4 mice from two independent experiments. Bars indicate mean ± SD. *P* values versus Prox1^+^ VS by two-tailed Mann-Whitney *U* test. **c**, **d**, Comparison of area and diameter of Prox1^+^ VS between male and female and between left and right nasal cavities in adult mice. Each dot indicates a value from one mouse and n = 8 mice/group from four independent experiments. Bars indicate mean ± SD. n.s., not significant *versus* Male or Left by two-tailed Mann-Whitney *U* test. Source data

Further analyses showed that ECs of the Prox1^+^ VSs were distinctive, unusual and a mixture of two types that were equally numerous. One type of intercellular junction was discontinuous, reflected by irregularly distributed VE-cadherin, and the other type was continuous, reflected by regularly distributed VE-cadherin (Fig. 1d and Extended Data Fig. 2a–g). In contrast, ECs of surrounding capillaries (diameter ∼10–15 μm) had tight intercellular junctions with several fenestrations (Fig. 1d and Extended Data Fig. 2a,e–g). Supporting these findings, plasmalemma vesicle-associated protein (PLVAP), a major molecule associated with fenestrations and trans-endothelial channels^25^, was abundant in the capillaries but rarely detectable in the Prox1^+^ VSs (Extended Data Fig. 3a,b). Prox1^+^ VSs were not positive for either vascular endothelial growth factor receptor 2 (VEGFR2) or VEGFR3, but the surrounding capillaries were VEGFR2^+^, and LVs were VEGFR3^+^ (Fig. 1e and Extended Data Fig. 3a). Moreover, Prox1^+^ VSs were covered by alpha-smooth muscle actin (αSMA)^+^ circular smooth muscle cells (SMCs), surrounded by PGP9.5^+^ peripheral nerve fibers and intermingled with mucin 5B^+^/glucose transporter 1 (GLUT1)^+^ mucosal glands (Fig. 1f). Iba1^+^ macrophages and major histocompatibility complex (MHC) II^+^ monocytic phagocytes and lesser amounts of CD3e^+^ T cells and B220^+^ B cells were distributed around Prox1^+^ VSs (Fig. 1g,h). These features did not differ by sex (male versus female mice) or by the side (left versus right nasal cavity) (Extended Data Fig. 3c,d).

### The nasal mucosa bears a mixture of typical and atypical LVs

**Fig. 2:**
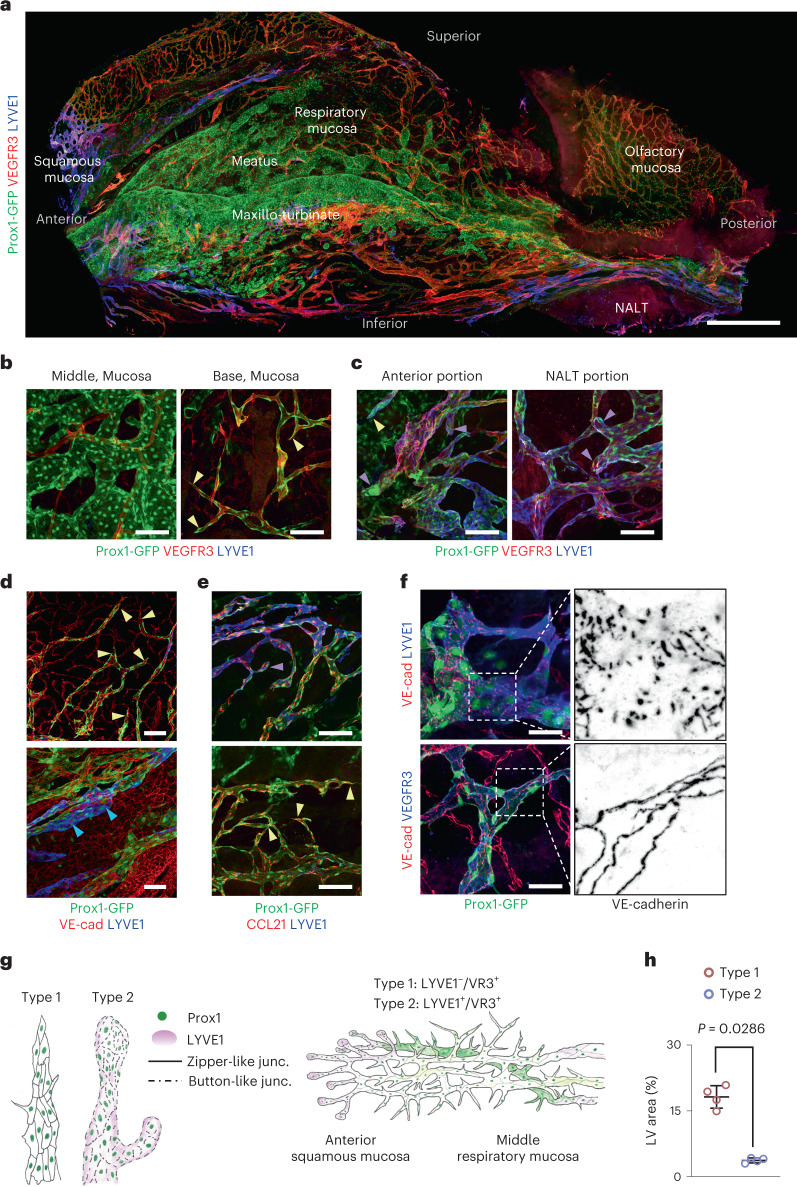
Distributions of typical and atypical LVs in the mouse nasal cavity. **a**, Image showing distributions of Prox1^+^/LYVE1^+^/VEGFR3^+^ LVs, Prox1^+^/LYVE1^−^/VEGFR3^+^ LVs and Prox1^+^ VSs at the indicated areas and regions of whole-mounted lateral side of nasal cavity of Prox1-GFP reporter mice. Scale bars, 500 μm. **b**–**e**, Images showing distributions of Prox1^+^ VSs at the middle portion and Prox1^+^/LYVE1^−^/VEGFR3^+^ LVs at the base portion of the meatus in the nasal mucosa (**b**), Prox1^+^/LYVE1^+^/VEGFR3^+^ LVs at anterior portion and around NALT (**c**) and LYVE1^+^/CCL21^+^ LVs and LYVE1^−^/CCL21^+^ LVs in the respiratory mucosa (**d**,**e**). Note the lymphatic capillary having sharp blunt-ends (light yellow arrowheads), lymphatic capillary having a round blunt-end (violet arrowheads) and LYVE1^+^ collecting LVs (blue arrowheads). VE-cad, VE-cadherin. Scale bars, 100 μm. **f**, Images showing button-like intercellular junctions (upper panels) in Prox1^+^/LYVE1^+^ LVs and zipper-like intercellular junctions (lower panels) in Prox1^+^/VEGFR3^+^ LVs. Scale bars, 50 μm. **a**–**f**, Similar findings were obtained from *n* = 6–8 mice per group from 3–5 independent experiments. **g**, Diagrams depicting two types of lymphatic capillary ends and distributions of two types of LVs in the nasal cavity. **h**, Comparison of the area between type 1 (LYVE1^−^/VEGFR3^+^) LVs and type 2 (LYVE1^+^/VEGFR3^+^) LVs in the nasal mucosa. Each dot indicates a value from one mouse and *n* = 4 mice from two independent experiments. Bars indicate mean ± s.d. *P* values versus type1 LVs by two-tailed Mann–Whitney *U-*test. Source data

**Extended Data Fig. 4:**
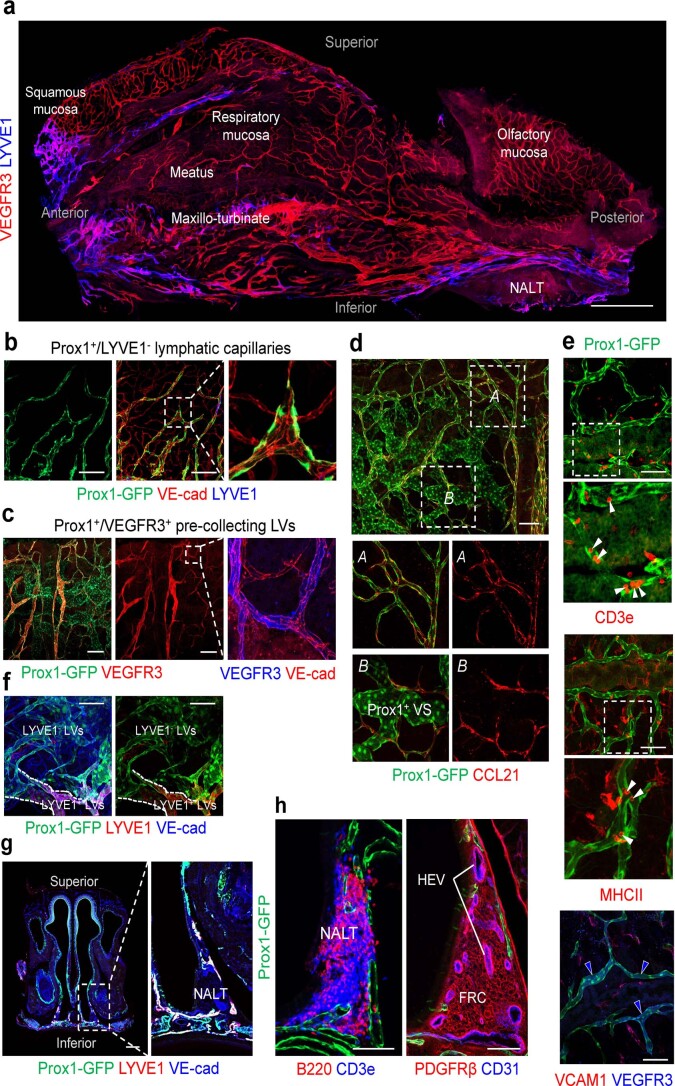
Distributions of typical and atypical LVs in the mouse nasal mucosa. **a**, Images showing distributions of LYVE1^+^/VEGFR3^+^ and LYVE1^−^/VEGFR3^+^ LVs at the indicated areas and regions of the whole-mounted lateral side of the nasal cavity of Prox1−GFP reporter mice. Scale bars, 500 μm. **b**, **c**, Images showing distributions of Prox1^+^/LYVE1^−^ lymphatic LVs and Prox1^+^/VEGFR3^+^ LVs. Each lined-box area is magnified and shown as the right panel. Scale bars, 200 μm. **d**, Images showing distributions of Prox1^+^/CCL21^+^ lymphatic LVs and Prox1^+^ VSs. Each alphabet area is magnified and shown as below panels. Scale bars, 200 μm. **e**, Images showing CD3e^+^ T cells (white arrowheads), MHCII^+^ cells inside LVs (white arrowheads), and no VCAM1 in Prox1^+^/VEGFR3^+^ LVs (blue arrowheads). Each lined-box area is magnified and shown as below panel. Scale bars, 100 μm. **f**, Images showing a connection (dotted lines) of Prox1^+^/LYVE1^−^ LVs and Prox1^+^/LYVE1^+^ LVs. Scale bars, 100 μm. **g**, Images showing distributions of Prox1^+^/LYVE1^+^ LVs in the cross-sectioned nasal cavity of Prox1-GFP reporter mice. Lined-box area is magnified and shown as the right panel. Scale bars, 500 μm. **h**, Images showing distributions of Prox1^+^ LVs in the cross-sectioned NALT area of Prox1-GFP reporter mice. B220^+^ B cells, CD3e^+^ T cells, CD31^+^ high endothelial venule (HEV) and PDGFRβ^+^ fibroblastic reticular cell (FRC) are shown. Scale bars, 100 μm. **a**–**h**, Similar findings were obtained from n = 4-6 mice from three to four independent experiments.

To distinguish Prox1^+^ LVs from Prox1^+^ VSs in the nasal cavity, we traced two specific lymphatic markers, LYVE1 and VEGFR3 (refs. ^12,26^). In addition to finding the LYVE1^+^/VEGFR3^+^ LVs as described above, we also identified LYVE1^−^/VEGFR3^+^ LVs thoroughly distributed in the respiratory mucosa (Fig. 2a and Extended Data Fig. 4a). Unlike most LYVE1^+^/VEGFR3^+^ lymphatic capillaries, which are rounded-ended and have button-like intercellular junctions^12,13,15,18^, the LYVE1^−^/VEGFR3^+^ LVs were sharp-ended and had zipper-like intercellular junctions (Fig. 2b–f and Extended Data Fig. 4b,c). Thus, two types of lymphatic capillaries are present in the nasal cavity: type 1, characterized by LYVE1^−^/VEGFR3^+^/Prox1^+^, sharp-ended and zipper-like lymphatic EC (LEC) junctions and mainly distributed in the respiratory mucosa; and type 2, characterized by LYVE1^+^/VEGFR3^+^/Prox1^+^, round-ended and button-like LEC junctions and primarily distributed in the squamous mucosa and around NALT (Fig. 2g,h and Extended Data Fig. 4).

All LECs occurring with LVs in the nasal cavity were chemokine (C-C motif) ligand (CCL)21^+^ and CCR7 ligand (Fig. 2e and Extended Data Fig. 4d). CD3e^+^ T cells or MHCII^+^ monocytic phagocytes were detected within the LVs (Extended Data Fig. 4e), implying that they constantly migrate to draining lymphoid tissues and back to the original place for keeping the immune surveillance of the nasal mucosa against constant pathogen invasion through inhaled air^27^. Both types of lymphatic capillaries were connected to LYVE1^−^/VEGFR3^+^/Prox1^+^ and LYVE1^+^/VEGFR3^+^/Prox1^+^ collecting LVs (Extended Data Fig. 4a–f). Nevertheless, we detected no connection between Prox1^+^ VSs and Prox1^+^ LVs, indicating a total separation of the blood circulation from the lymphatic drainage in the nasal cavity, consistent with other organs. The LVs, together with the capillaries, were mainly distributed in the submucosa (Fig. 2b,d and Supplementary Fig. 1a–c), which lies in the lower portion of the VS network.

Together, our 3D morphological and functional analyses in the murine nasal mucosa disclosed well-arranged vascular networks beneath a multi-layered epithelium in the following order: thin blood capillary plexus; multi-layered, large-caliber Prox1^+^ VSs; and a mixture of a dense blood capillary plexus and extensive lymphatic network, together with plenty of transverse submucosal glands (Supplementary Fig. 1d).

### Single-cell transcriptomic analysis shows distinct vascular heterogeneity in the nasal mucosa

**Fig. 3:**
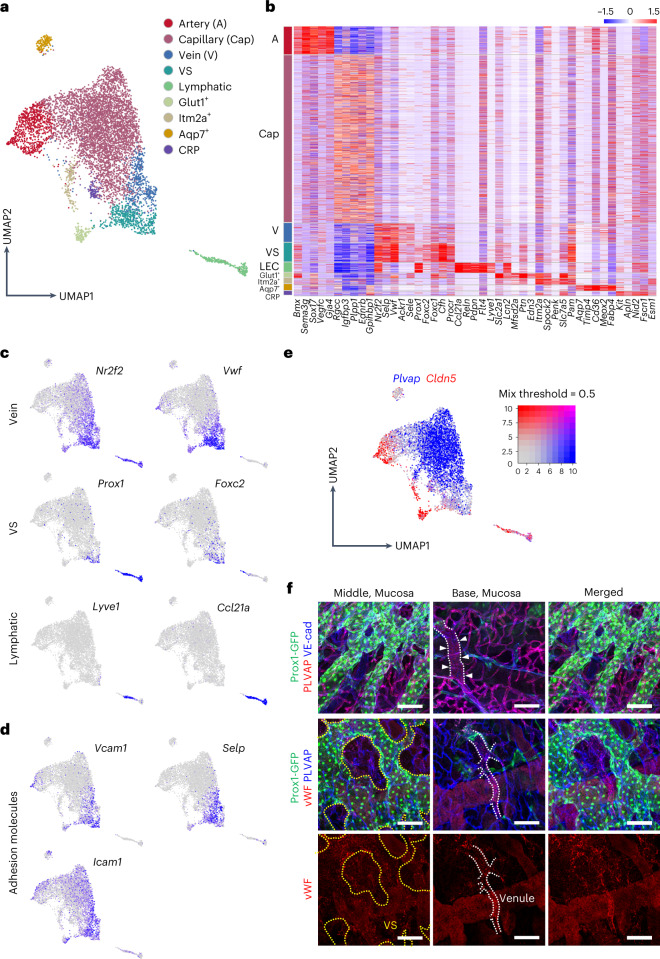
Single-cell transcriptomic analysis of vascular heterogeneity in the *tdTomato*^*rEC*^ mouse nasal mucosa. **a**, UMAP visualization of scRNA-seq data indicating nine clusters of ECs in the nasal cavity. **b**, Heat map visualizing distinct expression profiles of EC clusters. Scaled expression levels of five representative marker genes for each cluster are shown. **c**,**d**, UMAP plots showing normalized expression levels of indicated genes in the EC clusters of the nasal mucosa. **e**, UMAP plots showing blended scaled expression levels of *Plvap* (blue) and *Cldn5* (red). Note that the cells highly expressing both genes are color-blended (magenta). Palette shows cutoff percentiles for blending two colors in relative expression scales. Note that *Plvap* and *Cldn5* are exclusively expressed in the different EC clusters. **f**, Images showing distributions of PLVAP^−^/Prox1^+^ VS (yellow dotted lines) in the middle portion, PLAVP^+^/Prox1^−^ capillary in the base portion and PLAVP^+^/Prox1^−^/vWF^+^ venule (white dotted lines) in the base portion of the mouse nasal mucosa. Scale bars, 100 μm. Two independent experiments in *n* = 4 mice showed similar findings. Source data

**Extended Data Fig. 5:**
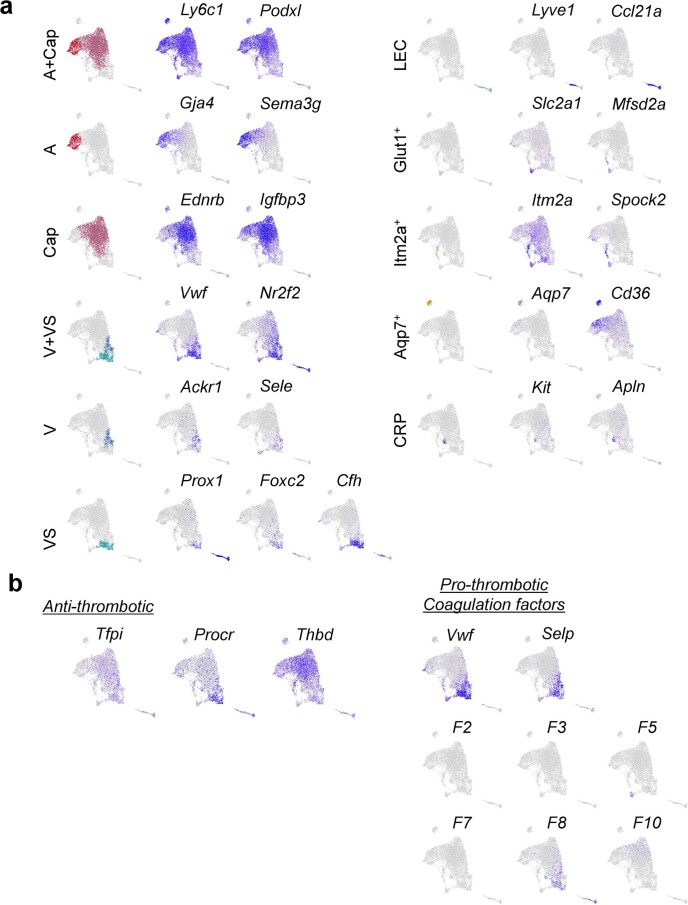
Distinct marker gene expressions distinguish heterogeneity of the mouse nasal vasculature and VS ECs highly express both anti-thrombotic and pro-thrombotic molecules. **a**, UMAP plots showing normalized expression levels of indicated marker genes that distinguish each EC cluster highlighted in the left panels. **b**, UMAP plots showing normalized gene expression levels of anti-thrombotic and pro-thrombotic molecules and coagulation factors.

To gain further insight into the nasal vasculature, we performed scRNA-seq on isolated cells from the mouse nasal mucosal tissue. Through unsupervised clustering, we identified a distinct EC cluster in the stromal cell clusters (Supplementary Fig. 2). This EC cluster was further classified into nine distinct subpopulations with specific enrichment, as follows: artery (*Gja4* and *Sema3g*), capillary (*Ednrb* and *Igfbp3*), vein (*Ackr1* and *Sele*), VS (*Foxc2* and *Cfh*), lymphatic (*Lyve1* and *Ccl21a*), Glut1^+^ (*Slc2a1* and *Mfsd2a*), integral membrane protein 2A (ITM2A)^+^ (*Itm2a* and *Spock2*), aquaporin 7 (AQP)7^+^ (*Aqp7* and *Cd36*) and capillary-resident regenerative population (CRP)^28^(*Kit* and *Apln*) (Fig. 3a,b and Extended Data Fig. 5a). Glut1^+^ ECs were distributed mainly in the dorsal meatus and septum regions of the nasal cavity (Supplementary Fig. 3a,b), and they distinctively expressed *Mfsd2a*, *Lcn2* and *Edn3* (Fig. 3b and Extended Data Fig. 5a), which are brain EC-specific transcripts^29,30^. Therefore, they may support or interact with the neural tissue of the nasal cavity. ITM2A^+^ ECs may be involved in the transcytosis of special molecules^31,32^. Moreover, we found not only a small population of Aqp7^+^ ECs that distinctively expressed the genes involved in the transport and metabolism of glycerol and fatty acids, such as *Cd36*, *Meox2* and *Fabp4* (refs. ^29,30^), but also a smaller population of the CRP ECs that distinctively expressed *Apln* and *Kit*^28^ (Fig. 3b and Extended Data Fig. 5a). In particular, the VS cluster differed from the vein cluster for the high enrichment in *Prox1* and *Foxc2*, and it was distinguished from the lymphatic cluster by a lack of *Lyve1* and *Ccl21a* (Fig. 3c). Moreover, VS cluster showed high enrichment in adhesion molecules, such as *Vcam1*, *Icam1* and *Selp* (Fig. 3d). We also noted that *Plvap* was highly enriched in the capillary cluster (Fig. 3e), consistent with IFS findings (Extended Data Fig. 3a,b and Fig. 3f). In contrast, *Cldn5* was highly enriched in the artery, ITM2A^+^, Glut1^+^ and lymphatic clusters (Fig. 3e). Accordingly, IFS for PLVAP and von Willebrand factor (vWF) clearly distinguished PLVAP^high^ capillary and PLVAP^+^/vWF^+^/Prox1^−^ vein and venule from PLVAP^−^/vWF^+^/Prox1^+^ VSs in the nasal mucosa (Fig. 3f).

**Extended Data Fig. 6:**
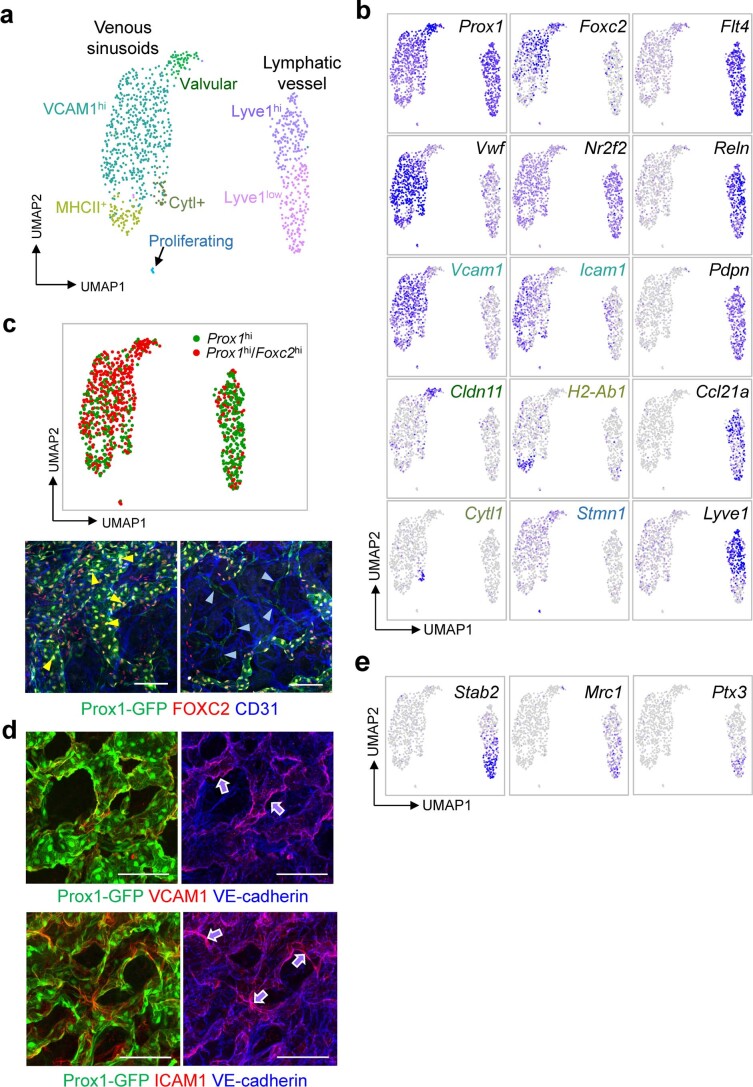
Plate-based scRNA-seq analysis of Prox1^+^ ECs in the Prox1-GFP mouse nasal mucosa. **a**, UMAP plots visualizing 5 clusters of VS ECs and 2 clusters of lymphatic ECs in the isolated Prox1^+^ ECs. **b**, UMAP plots showing expressions of indicated genes in the VS subclusters. **c**, UMAP plots showing *Prox1*^hi^/*Foxc2*^hi^ cell in the VS and lymphatic clusters, and images showing Prox1^+^/FOXC2^+^ ECs in VSs (yellow arrowheads) and Prox1^+^ ECs in LVs (light blue arrowheads). Scale bars, 100 μm. Similar findings were obtained from n = 5 mice from three independent experiments. **d**, Images showing VCAM^hi^ and ICAM1^hi^ in Prox1^+^ VSs (blue arrows). Scale bars, 100 μm. Similar findings were obtained from n = 5 mice from three independent experiments. **e**, UMAP plots showing enriched genes in the *Lyve1*^low^ subcluster.

To confirm these findings, we performed Smart scRNA-seq^33^ on isolated Prox1^+^ cells from the nasal mucosal tissue of Prox1-GFP reporter mice. Unsupervised clustering highlighted two major Prox1^+^ EC clusters: VS and lymphatic (Extended Data Fig. 6a). In the VS cluster, six distinct EC subpopulations were outlined with specific enrichment as follows: VCAM1^hi^ (*Foxc2, Nr2f2*, *Vwf*, *Vcam1* and *Icam1*), valvular (*Foxc2*, *Cldn11* and *Gja4*), cytokine like-1^+^ (*cytl1*), MHCII^+^ (*Cd74* and *H2-Ab1*) and proliferating (*Stmn1*) (Extended Data Fig. 6b and Supplementary Fig. 4). To our surprise, *Foxc2* was highly enriched in most ECs constituting the Prox1^+^ VSs cluster, which we confirmed using IFS (Extended Data Fig. 6c). We found that Foxc2, a mechanosensitive transcriptional factor that regulates differentiation and integrity of LECs and lymphatic valves^34–36^, was highly present in most Prox1^+^ VSs nuclei but not evident in nasal mucosa LVs (Extended Data Fig. 6c). The expression pattern of *Prox1* and *Foxc2* seemed unique to the nasal VSs because no transcripts of *Prox1* and *Foxc2* were detected in the sinusoidal ECs of bone marrow^37^ and liver^29^, although all three organs shared sinusoidal EC signatures (Supplementary Fig. 5). These findings suggest that Prox1 and Foxc2 may play important roles in maintaining nasal VS integrity. Nevertheless, a recent report^38^ indicates that Foxc2 and Prox1 play a preventive role for thrombosis in the venous valves by upregulating anti-thrombotic molecules while downregulating pro-thrombotic molecules, which led us to examine expression patterns of those molecules in the *Prox1*-enriched *a*nd *Foxc2*-enriched VS cluster. Among the transcripts regulating thrombosis and blood coagulation, *Procr* (anti-thrombotic molecule), *Vwf* and *Selp* (pro-thrombotic molecules) and *F8* (coagulation factor) were highly enriched in the VS cluster (Extended Data Fig. 5b), implying that Foxc2 and Prox1 may play important roles for preventing or enhancing thrombosis or coagulation at the nasal VSs in a context-dependent manner.

Consistent with the scRNA-seq data, IFS analysis showed high VCAM1 and ICAM1 in the ECs of Prox1^+^ VSs (Extended Data Fig. 6d). In comparison, the lymphatic cluster was distinguished from the VS cluster by high expression of *Flt4*, *Reln*, *Ccl21a* and *Pdpn* (Extended Data Fig. 6b and Supplementary Fig. 4). The lymphatic cluster was further classified into two distinct subpopulations, Lyve1^high^ and Lyve1^low^ (Extended Data Fig. 6a,b), which coincided with two types of nasal mucosa LVs: LYVE1^+^/VEGFR3^+^/Prox1^+^ and LYVE1^−^/VEGFR3^+^/Prox1^+^ LVs (Fig. 2 and Extended Data Fig. 4). *Stab2*, *Mrc1* and *Ptx3* were selectively and highly enriched in the Lyve1^low^ lymphatic cluster (Extended Data Fig. 6e), implying that LYVE1^−^/VEGFR3^+^/Prox1^+^ LVs may be involved in antigen presentation and immune modulation in the nasal mucosa. Thus, the molecular landscape of vascular heterogeneity in the nasal cavity is distinct from the vascular heterogeneity of other organs.

### Identification of upstream and downstream connections of Prox1^+^ VSs

**Fig. 4:**
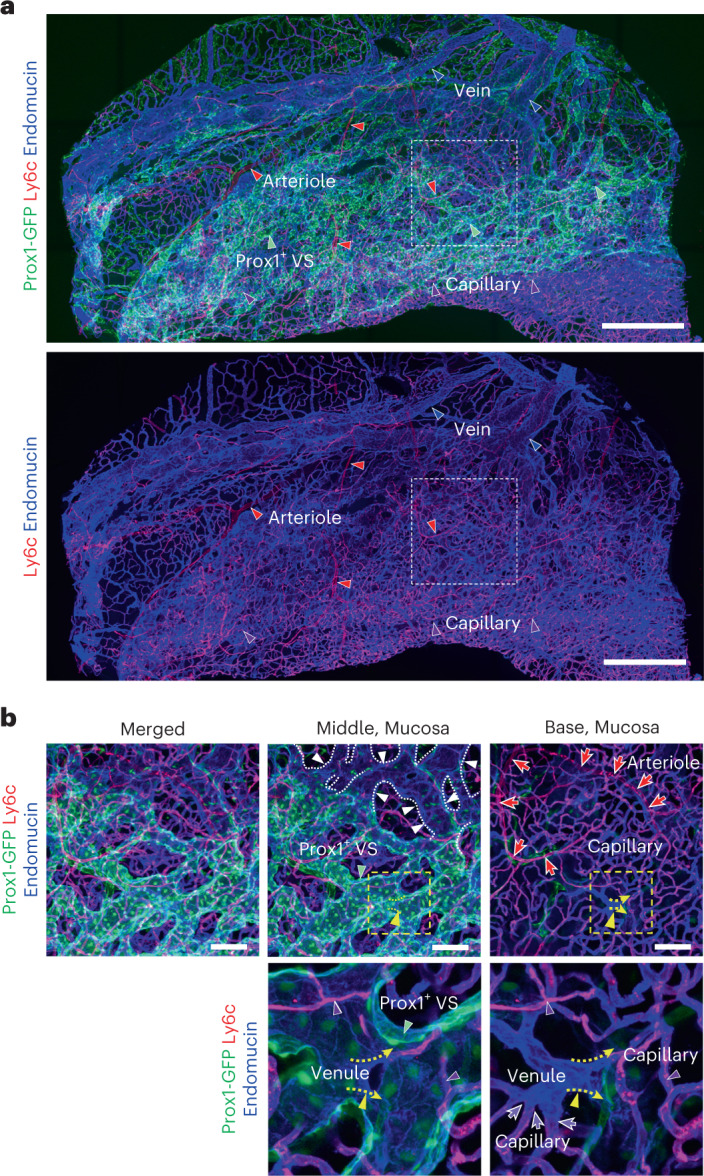
Immunolocalization reveals unique vascular connections in the mouse nasal mucosa. **a**,**b**, Images showing immunolocalization of nasal vasculature by simultaneous i.v. injection of fluorescence-labeled anti-Ly6c (for detection of artery, arteriole and capillary) and anti-endomucin (for detection of capillary and vein) antibodies to Prox1-GFP reporter mice. Scale bars, 500 μm (**a**) and 100 μm (**b**). White dashed-lined boxes in **a** are magnified and presented in **b**. Arterioles (red arrowheads and arrows; Ly6c^+^/endomucin^−^), capillaries (purple arrowheads and arrows; Ly6c^+^/endomucin^+^), venules (yellow arrowheads and arrowhead lines; Prox1^−^/endomucin^+^/Ly6c^−^), VSs (green arrowheads; Prox1^+^/endomucin^+^/Ly6c^−^) and veins (blue arrowheads, white arrowheads and dotted lines; Prox1^−^/endomucin^+^/Ly6c^−^). The Ly6c^+^/endomucin^+^ capillaries merge into the endomucin^+^ venule that is connected to the endomucin^+^/Prox1^+^ VSs. Similar findings were obtained from *n* = 3 mice from two independent experiments. Source data

**Extended Data Fig. 7:**
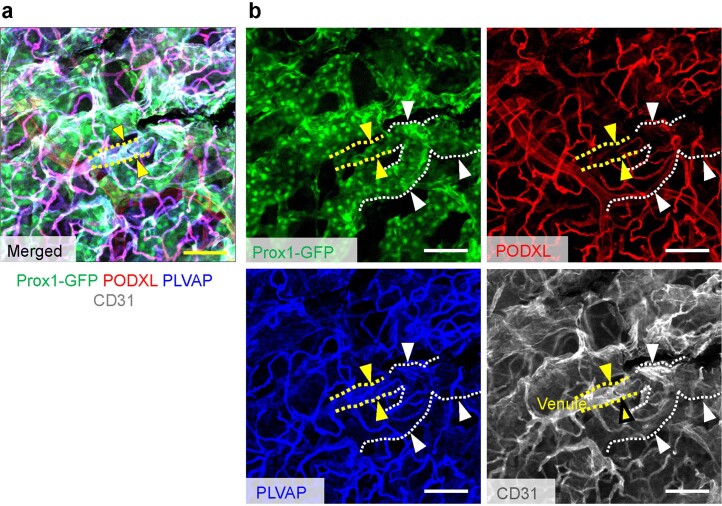
Immunolocalization reveals unique vascular connections in the mouse nasal mucosa. **a**, Images showing immunolocalization of nasal vasculature by simultaneous i.v. injection of fluorescence-labeled anti-podocalyxin (PODXL) (for detection of arterioles and capillary), anti-PLVAP (for detection of capillary and vein), and anti-CD31 (all vessels) antibodies to Prox1-GFP reporter mice. Scale bars, 100 μm. **b**, Four different single-channel images from the merged channel image of **a** are shown. Note that venule (yellow arrowheads and yellow lines; Prox1^-^/PODXL^−^/PLVAP^+^/CD31^+^) is located between capillaries (Prox1^−^/PODXL^+^/PLVAP^+^/CD31^+^) and VSs (white arrowheads and white lines; Prox1^+^/ PODXL^−^/PLVAP^−^/CD31^+^). Scale bars, 100 μm. Two independent experiments in n = 3 mice show similar findings.

According to a previous report^28^ and our scRNA-seq data, we determined upstream and downstream connections of Prox1^+^ VSs by imaging whole nasal vasculature removed shortly after intravenous (i.v.) administration of fluorescence-labeled antibodies to Prox1-GFP reporter mice as follows: anti-Ly6c and anti-podocalyxin (PODXL), which stain for arteries and capillaries; anti-endomucin and anti-PLVAP, which stain for capillaries and veins but not arteries; and anti-CD31, which stains for whole blood vessels. This approach allowed us to identify the vascular connections within the whole nasal vascular tree, from arteries and arterioles (anti-Ly6c and anti-PODXL labeled), to capillaries (anti-Ly6c, anti-PODXL, anti-endomucin and anti-PVAP labeled), venules (anti-endomucin and anti-PLVAP labeled), Prox1^+^ VSs (anti-endomucin labeled) and veins (anti-endomucin labeled) (Fig. 4, Extended Data Fig. 7, Supplementary Video 1 and Supplementary Fig. 6). We especially found that the Ly6c^+^/PODXL^+^/endomucin^+^/PLVAP^+^ capillaries merged into the endomucin^+^/PLVAP^+^ venules that were connected to the endomucin^+^/Prox1^+^ VSs (Fig. 4, Extended Data Fig. 7, Supplementary Video 1 and Supplementary Fig. 6). Nevertheless, the approaches used did not enable us to identify arteriovenous anastomoses, which are likely to be present, based on the reports of others^1–4,9,10^.

### Human nasal VSs are PROX1^+^/FOXC2^+^ and *VCAM1*^high^

**Fig. 5:**
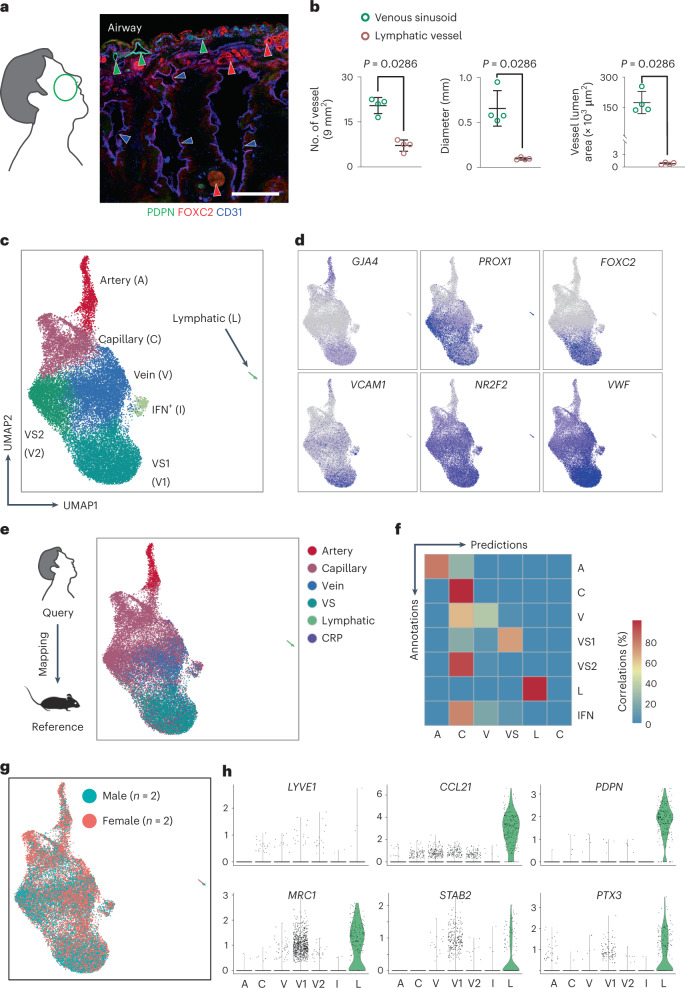
PROX1^+^ VSs and LVs in the human nasal mucosa. **a**,**b**, Diagram showing the tissue sampling of human nasal mucosa and images and quantification of FOXC2^+^/CD31^+^ VS (blue arrowheads) and PDPN^+^/CD31^+^ LVs (green arrowheads). The anti-FOXC2 antibody produces a non-specific positive signal in the mucosal glands (red arrowheads), presumably because of the mucus’s stickiness. Scale bar, 500 μm. Each dot in **b** indicates a value from each patient and *n* = 4 patients. Bars indicate mean ± s.d. *P* values versus VS by two-tailed Mann–Whitney *U-*test. **c**, UMAP plot visualizing heterogeneity of EC subpopulations in the human nasal mucosa. **d**, UMAP plot depicting normalized expression levels of *GJA4*, *PROX1*, *FOXC2*, *VCAM1*, *NR2F2* and *VWF* in the indicated clusters. **e**, Diagram and UMAP plot showing the reference mapping of human nasal ECs using mouse nasal ECs as a reference. **f**, Heat map showing degrees of correlations between the annotations and the reference mapping in **e**. **g**, UMAP plot visualizing similar EC heterogeneity between male and female patients (each *n* = 2). **h**, Violin plots showing exclusive expression of indicated genes in lymphatic EC cluster. Source data

To examine whether the patterns in mice are conserved in humans, we performed IFS and scRNA-seq analyses on normal portions of human nasal mucosal tissues obtained from four patients (two females and two males) with severe deviated nasal septum during the inferior turbinoplasty (Supplementary Table 1). Similarly to the morphological features of the mouse nasal VSs and LVs, human nasal VSs were extensively distributed, had relatively large vascular diameter and lumen and were PROX1^+^/FOXC2^+^, whereas human nasal LVs were less distributed, had relatively small vascular diameter and lumen and were PDPN^+^/PROX1^+^ (Fig. 5a,b). Among seven distinct EC clusters derived from the pooled ECs of the four patients, we noted two types of PROX1^+^ VS clusters, distinguished by selective or higher expression of *FOXC2*, *VCAM1* and *VWF* in VS1 versus VS2 (Fig. 5c,d). To refine the similarity and difference between human nasal ECs and mouse nasal ECs, we performed reference mapping and annotations between two species of nasal ECs (Fig. 5e). Human VS1 was similar to mouse VS, whereas human VS2 was different from mouse VS in not expressing *FOXC2* but expressing *PROX1* (Fig. 5f). It would be interesting to delineate the differences between human VS1 and VS2. Moreover, the EC heterogeneity between male and female patients was indifferent (Fig. 5g). Furthermore, PROX1^+^ LECs constituting nasal LVs had low *LYVE1* expression but high expression of *CCL21*, *PDPN*, *MRC1*, *STAB2* and *PTX3* (Fig. 5h), which are involved in immune cell recruitment and transmigration, antigen presentation and immune modulation^13,39,40^. These findings indicate that the morphological and molecular landscapes of the nasal mucosal vasculature are largely conserved between mouse and human.

### Nasal VSs are contracted in an AR model

**Fig. 6:**
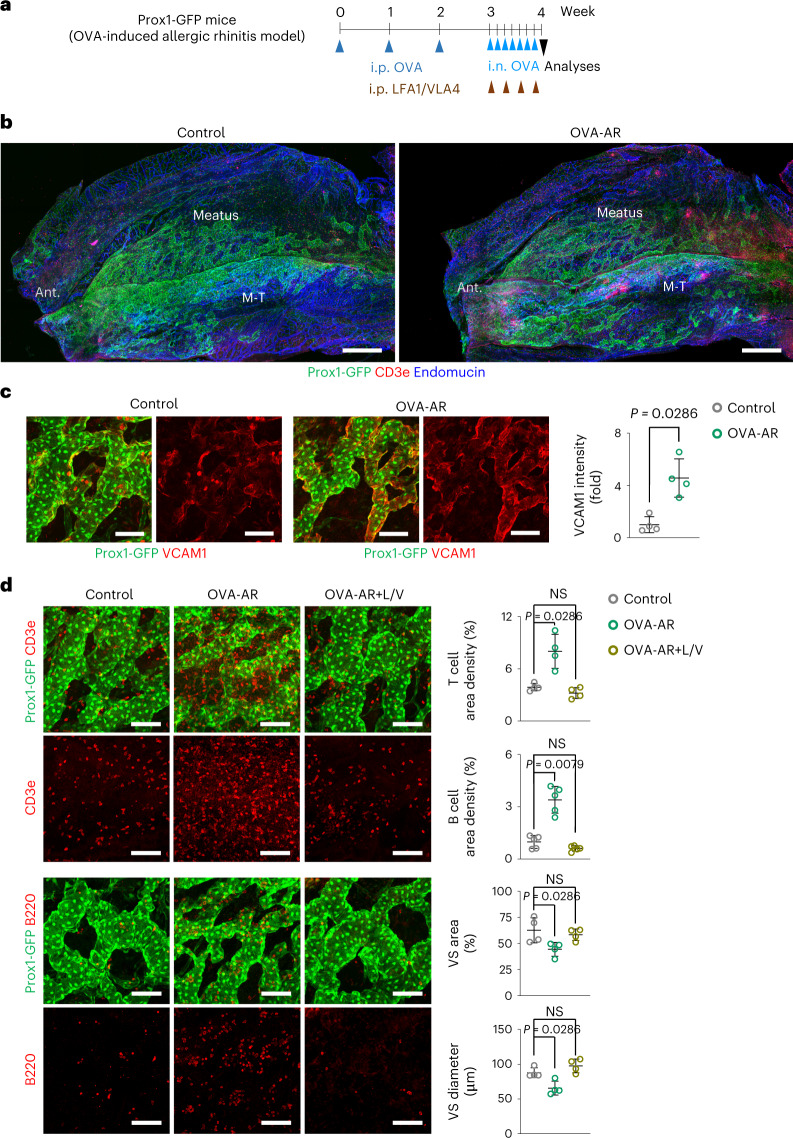
OVA-AR mouse model shows VS contraction and increased lymphoid cell infiltrations in the nasal mucosa. **a**, Diagram depicting the generation of OVA-AR model in adult Prox1−GFP mice by i.p. administrations of OVA (1-week interval) followed by i.n. administrations of PBS or OVA (1-day interval). i.p. administration of PBS or the cocktail blocking antibodies against LFA1 and VLA4 (L/V) was performed from the starting day of i.n. administration of OVA. **b**, Images showing distributions of Prox1^+^ VSs, endomucin^+^ capillaries and CD3e^+^ T cells of whole-mounted lateral side nasal cavity of Prox1-GFP reporter mice. M-T, maxillo-turbinate. Note reduced area and diameter of Prox1^+^ VSs in the OVA-AR group. Scale bars, 500 μm. Three independent experiments in *n* = 4 mice showed similar findings. **c**, Images and comparison of VCAM1 in the Prox1^+^ VSs. Scale bars, 100 μm. VCAM1 intensity was normalized on the control intensity values. Each dot indicates a value from one mouse and *n* = 4 mice per group from three independent experiments. Bars indicate mean ± s.d. *P* values versus control by two-tailed Mann–Whitney *U-*test. **d**, Images and comparisons of distributions of CD3e^+^ T cells and B220^+^ B cells and area and diameter of Prox1^+^ VSs. Scale bars, 100 μm. Each dot indicates a value from one mouse and *n* = 4–5 mice per group from three independent experiments. Bars indicate mean ± s.d. *P* values versus control by two-tailed Mann–Whitney *U-*test. NS, not significant. Source data

**Fig. 7:**
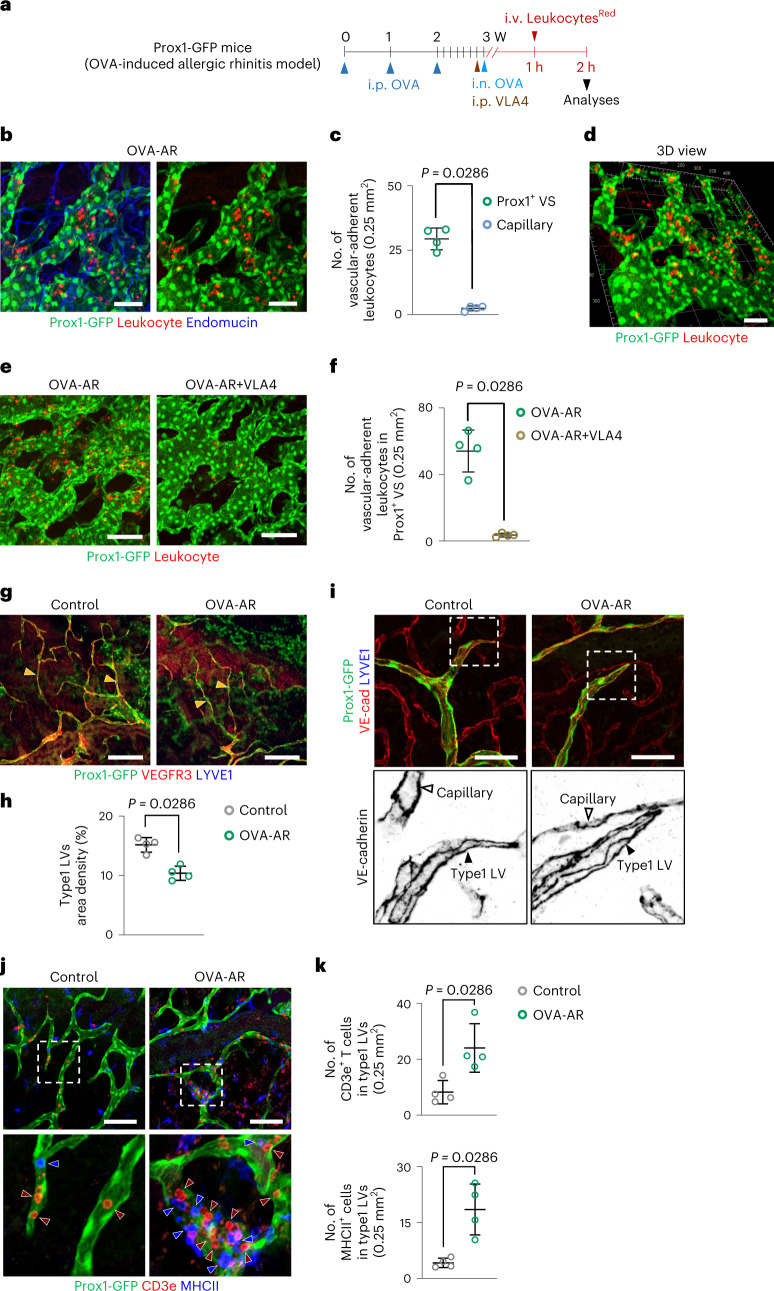
Prox1^+^ VS serves as a major vessel for leukocyte trafficking in the nasal vasculature, and OVA-AR induces regression of type 1 LVs but increases immune cell infiltration within the LVs. **a**, Diagram depicting the generation of OVA-AR model in adult Prox1-GFP by i.p. and i.n. administrations of OVA. i.p. administration of blocking antibody against VLA4 was performed. In total, 1 × 10^7^ of Leukocytes^Red^ was administered through the tail vein 1 hour after the i.n. OVA administration. **b**,**c**, Images and comparison of the number of vascular-adherent Leukocytes^Red^ between Prox1^+^ VS and endomucin^+^ capillary. Scale bars, 50 μm. Each dot indicates a value from one mouse and *n* = 4 mice from two independent experiments. Bars indicate mean ± s.d. *P* values versus Prox1^+^ VS by two-tailed Mann–Whitney *U-*test. **d**, 3D image showing localization of vascular-adherent Leukocytes^Red^ within Prox1^+^ VS. Scale bar, 50 μm. **e**,**f**, Images and comparison of the number of vascular-adherent Leukocytes^Red^ in Prox1^+^ VS between OVA-AR and OVR-AR + VLA4. Scale bars, 100 μm. Each dot indicates a value from one mouse and *n* = 4 mice per group from two independent experiments. Bars indicate mean ± s.d. *P* values versus OVA-AR by two-tailed Mann–Whitney *U-*test. **g**,**h**, Images and comparison of the density of LYVE1^−^/VEGFR3^+^ type1 LVs (yellow arrowheads) in the nasal mucosa between control and OVA-AR mice. Scale bars, 200 μm. Each dot indicates a value from one mouse and *n* = 4 mice per group from two independent experiments. Bars indicate mean ± s.d. *P* values versus control by two-tailed Mann–Whitney *U-*test. **i**, Representative images showing no change of the zipper-like EC junction in type 1 LVs between control and OVA-AR mice. Scale bars, 50 μm. Two independent experiments in *n* = 4 mice showed similar findings. **j**,**k**, Images and comparisons of CD3e^+^ T cells (dark-red arrowheads) and MHCII^+^ cells (blue arrowheads) within type1 LVs in the nasal mucosa between control and OVA-AR mice. Scale bars, 100 μm. Each dot indicates a value from one mouse and *n* = 4 mice per group from two independent experiments. Bars are mean ± s.d*. P* values versus control by two-tailed Mann–Whitney *U-*test. Source data

VCAM1 is a major adhesion molecule responsible for leukocyte trafficking from the vascular lumen to the interstitial compartment^41^. Because most Prox1^+^ VS ECs also had high VCAM1, we investigated how the VSs respond to chronic inflammation. AR is one of the most common inflammatory diseases affecting the human nasal cavity^42^. To recapitulate chronic inflammation in the nasal cavity, we adapted an ovalbumin-induced AR (OVA-AR) mouse model^43^ (Fig. 6a). At 1 week after initial nasal induction of OVA, we found a substantial increase in VCAM1 with increased infiltration of CD3e^+^ T cells and B220^+^ B cells in the nasal mucosa (Fig. 6b–d). To address the role of VCAM1 in Prox1^+^ VSs in AR pathogenesis, we treated the OVA-AR mice with a cocktail of blocking antibodies against lymphocyte function-associated antigen 1 (LFA1) and very late antigen 4 (VLA4, integrin α4β1) (intraperitoneal (i.p.), 50 mg kg^−1^ of body weight each, four times at 2-day intervals) for eight consecutive days after the initial nasal induction of OVA. The treatment almost completely inhibited the increased infiltration of CD3e^+^ T cells and limited the reductions in Prox1^+^ VS area and diameter (Fig. 6d), indicating that increased VCAM1 in Prox1^+^ VS could be key to AR progression. To address whether Prox1^**+**^ VS is primarily responsible for the increased infiltration of the immune cells, we isolated leukocytes from the mouse spleen, labeled them with a Red-CMPTX fluorescence dye (we designate these cells as ‘Leukocytes^Red^’) and i.v. administered Leukocytes^Red^ to the mice, which had been subjected to the first challenge of intra-nasal OVA 1 hour before (Fig. 7a). At 1 hour after the i.v. administration of Leukocytes^Red^, the number of vascular-adherent Leukocytes^Red^ was 11.0-fold higher in Prox1^**+**^ VSs than in other surrounding vessels (Fig. 7b–d). Of note, Leukocytes^Red^ adhesion was almost completely inhibited by the anti-VLA4 antibody pre-treatment (Fig. 7e,f). Based on these findings, we regard Prox1^+^ VSs as a primary site of leukocyte recruitment through the increased VCAM1 in the inflammatory condition.

To our surprise, however, Prox1^+^ VS area and diameter were reduced in the nasal mucosa of OVA-AR mice (Fig. 6b–d). To assess further the basis of reduced Prox1^+^ VSs in OVA-AR mice, we performed scRNA-seq analysis on nasal mucosa samples from the control and OVA-AR mice. As expected, the proportion of immune cells among the four main clusters (stromal, neuronal, immune and epithelial) increased from 20% in controls to 49% in OVA-AR mice (Supplementary Fig. 7a–c). Of these changes, the proportions of B cells, T cells and monocytes predominantly increased (Supplementary Fig. 7d). Among T cells, the proportion of CD4^+^ T cells increased from 0.3% in controls to 31.8% in OVA-AR mice, whereas proportions of other types of T cells declined or remained relatively unchanged (Supplementary Fig. 7e,f). Expression of interferon-γ, a strong vascular regression cytokine^44,45^, was highly increased in clusters of natural killer type 2 (Nk2) cells and type 2 innate lymphoid cells (ILC-2) (Supplementary Fig. 7g). Thus, the VS reduction could be attributed to a marked increase in CD4^+^ T cells and interferon-γ.

We also noted that the area of type 1 LVs was reduced by 34% with preserving the zipper-like intercellular junction in OVA-AR mice (Fig. 7g–i). Nevertheless, the numbers of infiltrated CD3e^+^ T cells and MHCII^+^ monocytic phagocytes within type 1 LVs increased 3.1-fold and 4.9-fold in OVA-AR mice compared to control mice (Fig. 7j,k). These findings imply that type 1 LVs serve as a transmigration conduit for the immune cells from the nasal mucosa to the draining lymphoid tissues not only under normal conditions but also under inflammatory conditions.

**Extended Data Fig. 8:**
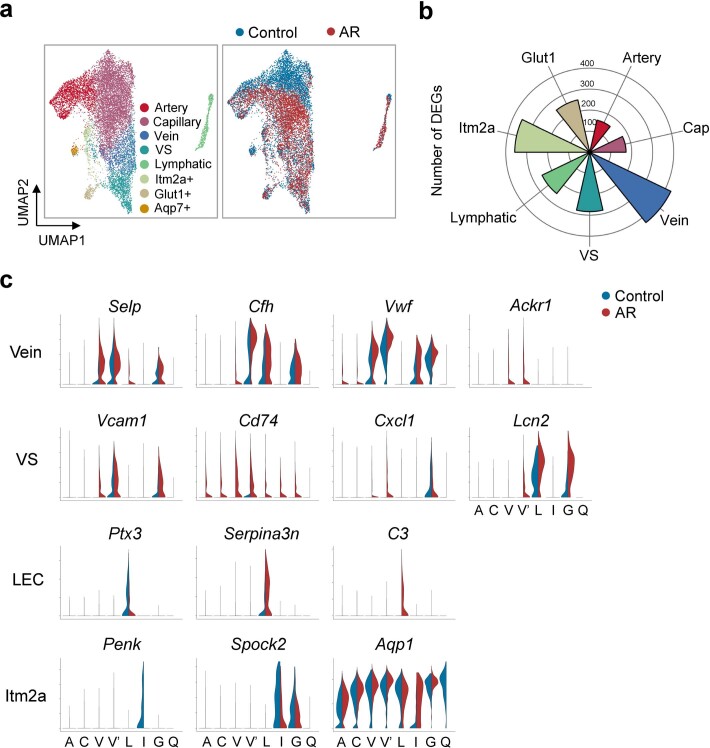
scRNA-seq analysis reveals changes of expression profiles of the key genes in the EC subpopulations of the nasal mucosa by an allergic rhinitis induction. **a**, UMAP plot visualizing the subpopulations constituted the pooled ECs derived from the nasal mucosa of Control and AR. **b**, Rose plot showing the number of differentially altered genes in the indicated cluster in AR compared to Control. **c**, Violin plot depicting differential expression of indicated genes in indicated clusters between Control and AR. A, artery; C, capillary; V, vein; V’, venous sinusoids; L, lymphatic; I, Itm2a^+^ EC; G, Glut1^+^; Q, Aqp7^+^.

Further scRNA-seq analyses revealed eight subpopulations of ECs, derived from the pooled ECs of control and OVA-AR mice (Extended Data Fig. 8a). The numbers of differentially expressed genes between the two groups were dominant in the vein, VS and Itm2a clusters (Extended Data Fig. 8b). In these clusters, we compared highly expressed genes related to vascular inflammation between control and OVA-AR mice, visualized in violin plots (Extended Data Fig. 8c). *Selp*, *cfn*, *Vwf*, *Ackr1*, *Vcam1*, *Cd74*, *Cxcl1* and *Lcn2* were upregulated in the vein and VS clusters, whereas *Ptx3* was downregulated but *serpina3n* and *C3* were upregulated in the lymphatic cluster (Extended Data Fig. 8c). Of note, *Penk* and *Spock2* were downregulated but *Aqp1* was upregulated in the Itm2a EC cluster (Extended Data Fig. 8c). These findings imply that each EC cluster in the nasal vasculature distinctly and uniquely responds to AR.

### SARS-CoV-2 infection of the nasal epithelium compromises nasal VSs

**Extended Data Fig. 9:**
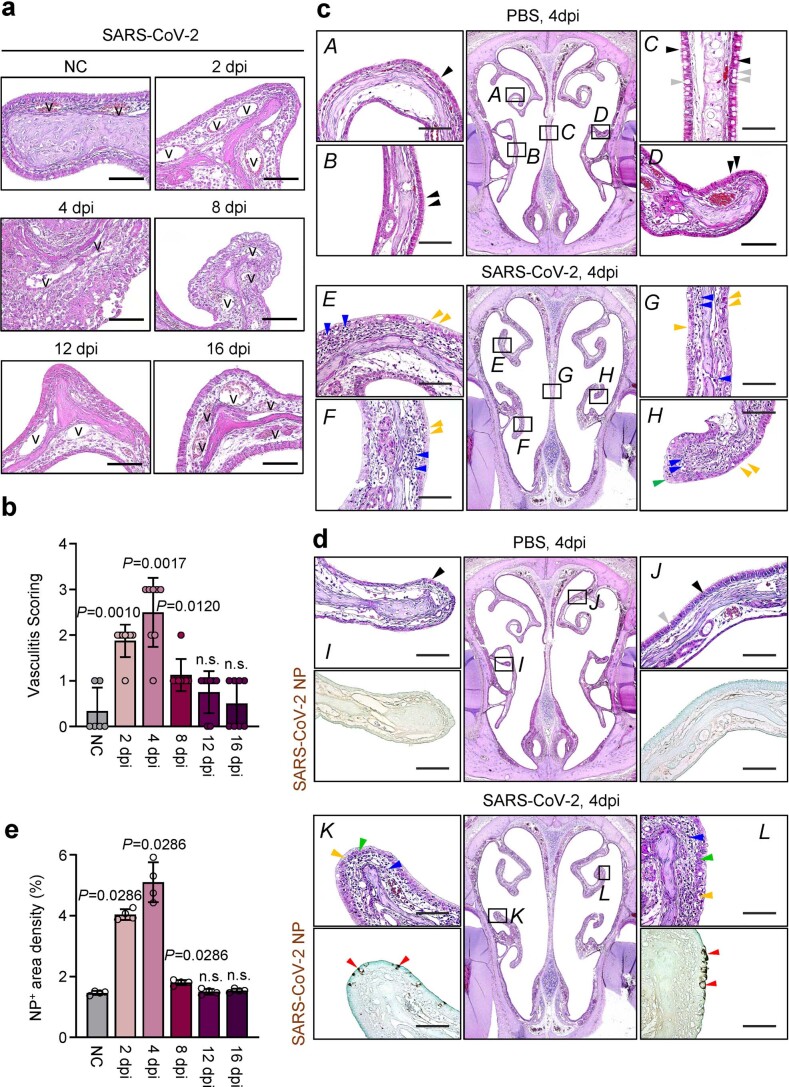
Nasal VSs is compromised in the COVID-19 hamster model. **a**, **b**, Histopathological changes in the nasal turbinate (NT) following days post-infection (dpi) of SARS-CoV-2 into the nasal cavity of hamster. Cross sections of the NTs are H&E stained. The lesions are started to be detected from 2 dpi, peaked at 4 dpi, and then gradually recovered until 16 dpi. NC, non-infected control; V: venous sinusoids. Scale bars, 50 μm. Vasculitis scoring at indicated dpi of SARS-CoV-2 infection. Each dot indicates a value from one mouse, and n = 6 for NC group and n = 8 mice for other groups from two independent experiments. Vertical bars indicate mean ± SD. *P* values versus NC by two-tailed Mann-Whitney *U* test. n.s., not significant. **c**, Representative H&E stained images showing comparisons of microscopic lesions between PBS-treated and SARS-CoV-2-infected nasal cavity of the hamster at 4 dpi. Intact ciliated pseudostratified columnar epithelium (black arrowheads) with goblet cells (gray arrowheads) are seen in the PBS-treated subject, while damaged squamous epithelium with vacuolation (yellow arrowheads), hyperplastic changes (green arrowhead), and infiltration of inflammatory cells into the lamina propria and submucosa (blue arrowheads) are seen in the SARS-CoV-2-infected subject. *A* and *E*, dorsal nasoturbinates; *B*, *D*, *F*, and *H*, ventral nasoturbinates; *C* and *G*, nasal septum. Scale bars, 50 μm. Similar findings were obtained from n = 7-8 mice/group from three independent experiments. **d, e**, Representative H&E stained and IHC images and comparison of SARS-CoV-2 nucleocapsid protein (NP) between PBS-treated and SARS-CoV-2-infected nasal cavity of the hamster at 4 dpi. No NP is detected in PBS-treated subject, while strong NP (red arrowheads) is detected in the epithelium. Black arrowheads, intact ciliated pseudostratified columnar epithelium; gray arrowhead, goblet cell; yellow arrowheads, damaged epithelium with vacuolation; green arrowheads, hyperplastic changed epithelium; blue arrowheads, inflammatory cell infiltration. *I* and *K*, ventral nasoturbinates; *J* and *L*, dorsal nasoturbinates. IHC images are counterstain with methyl green. Scale bars, 50 μm. Quantifications of NP^+^ area in the nasal turbinates at the indicated dpi of SARS-CoV-2 infection. NC, non-infected control. Each dot indicates a value from one mouse and n = 4 mice/group from two independent experiments. Vertical bars indicate mean ± SD. *P* values versus NC by two-tailed Mann-Whitney *U* test. n.s., not significant. Source data

Multi-ciliated cells of the nasal mucosal epithelium are the primary target of SARS-CoV-2 infection^19,20^, which leads to infiltration of immune cells and dysregulated inflammatory cytokine secretion, with the possible compromise of surrounding tissues during the early stage of COVID-19 (refs. ^19,20^). Moreover, patients with COVID-19 show vascular abnormalities, including hypercoagulation and endothelial dysfunction, which implicate the vasculature in COVID-19 pathogenesis^46,47^. To examine whether COVID-19 involves compromised nasal VSs, we generated an animal model of COVID-19 by inoculation of SARS-CoV-2 into the nasal cavity of the Syrian hamster, as previously reported^48^. At 2 days post-infection (dpi), the nasal turbinate had developed vacuolized and cilia-deficient epithelial cells, largely disrupted epithelium lining, dilated VSs and infiltrations of inflammatory cells, including neutrophils, lymphocytes and mononuclear cells, into the mucosa, lamina propria and submucosa and around the VSs (Extended Data Fig. 9a,b). At 4 dpi, inflammation severity and the number of SARS-CoV-2 nucleocapsid-protein-positive epithelial cells peaked, and the inflammatory cells were detected mainly in the dilated VSs (Extended Data Fig. 9a–e). This inflammation gradually subsided until 16 dpi, and the nucleocapsid-protein-positive epithelial cells were no longer detectable from 8 dpi (Extended Data Fig. 9a,b,e). Nevertheless, no SARS-CoV-2 nucleocapsid protein was detected in any cell type of the nasal vasculature, including ECs, which is consistent with previous reports^49^. Thus, nasal VSs could be heavily compromised by inflammatory cell infiltration induced by the battery of cytokines secreted from nasal epithelial cells infected by SARS-CoV-2 (refs. ^46,47^).

### Prox1^+^ VSs emerge from nascent blood vessels during embryonic development

**Extended Data Fig. 10:**
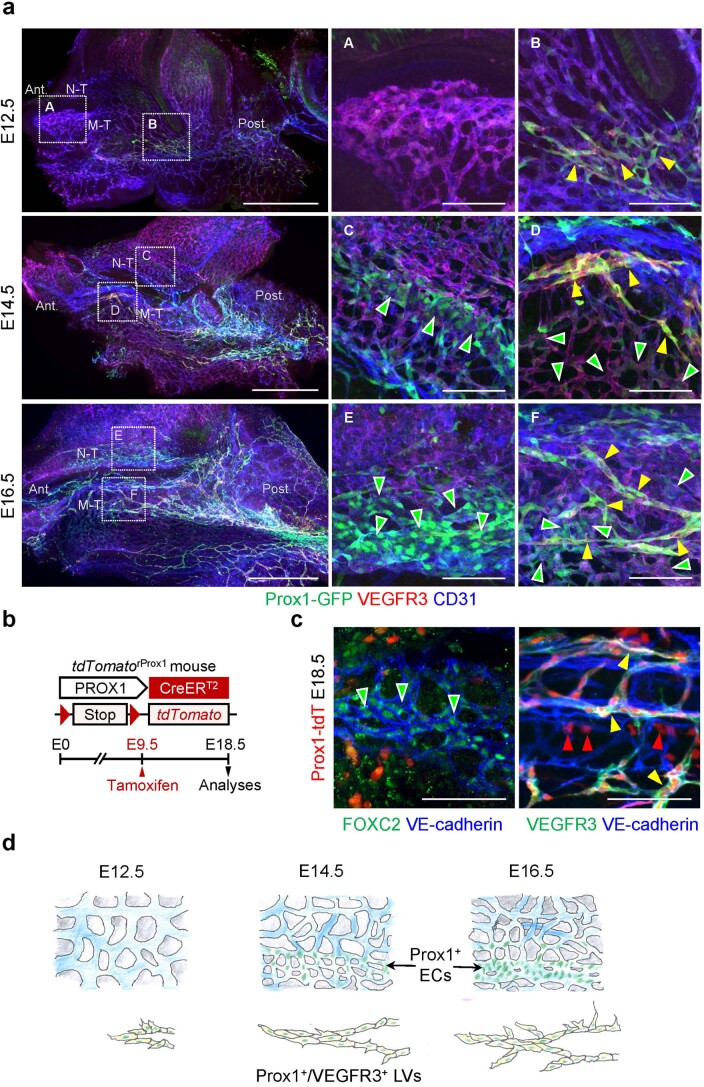
Prox1^+^ VS emerges at E14.5 in mice. **a**, Images of vasculatures in the nasal cavity of Prox1-GFP reporter mice at E12.5, E14.5 and E16.5 (Scale bars, 500 μm). Each large alphabet area is magnified in the middle and right panels (Scale bars, 100 μm). Yellow arrowheads indicate growing VEGFR3^+^ LVs, while green arrowheads indicate emerging and maturating Prox1^+^ VS. Ant., anterior portion; Post., posterior portion; N-T, nasoturbinate; M-T, maxilloturbinate. Similar finding was shown from n = 4 mice/group from two independent experiments. **b**, **c**, Diagram depicting generation of *tdTomato*^rProx1^ mouse and experimental scheme for the lineage tracing assay. Tamoxifen administration at E9.5 and sampling at E18.5. Representative images showing no Prox1-tdTomato (tdT) signal in the FOXC2^+^ VS (green arrowheads) but apparent Prox1-tdT signal in the VEGFR3^+^ LVs (yellow arrowheads) of nasal vasculatures at E18.5. Prox1-tdT^+^/VEGFR3^−^ cells (red arrowheads) are likely tuft cells in the respiratory epithelium. Scale bars, 100 μm. Similar finding was shown from n = 4 mice/group from two independent experiments. **d**, Diagram depicting Prox1^+^ VS emergence and Prox1^+^/VEGFR3^+^ LV formation in the nasal cavity of developing mouse embryos.

To uncover how Prox1^+^ VSs originate, we examined the spatiotemporal distributions of Prox1^+^ vessels in the nasal cavity during the embryonic development of Prox1-GFP reporter mice. At embryonic day 12.5 (E12.5), a vessel characterized by Prox1^+^ ECs was not detected in the putative VS region of the nasal cavity, but a fair amount of Prox1^+^ ECs composing the growing LVs was present in the posterior nasal cavity (Extended Data Fig. 10a). The latter LVs were connected with those of the neck and upper thoracic cavity (Extended Data Fig. 10a). At E14.5, Prox1^+^/FOXC2^+^/VEGFR3^low^ ECs had emerged in the CD31^+^ nascent vasculature at the turbinate regions, and the growing Prox1^+^/VEGFR3^high^ LVs had expanded into the nasal cavity (Extended Data Fig. 10a). The ECs constituting putative Prox1^+^ VSs were VEGFR2^+^, Tie2^+^, VEGFR3^low^ and FOXC2^+^ at E14.5 (Supplementary Fig. 8a,b). At E16.5 and E18.5, the ECs of immature Prox1^+^ VSs showed distinct Prox1 and FOXC2 expression and were VEGFR2^low^, Tie2^high^ and VEGFR3^low^ (Extended Data Fig. 10a and Supplementary Fig. 8a,b). These findings imply that Prox1^+^ VSs originate from the nascent blood vessels at around E14.5.

To confirm this origin of Prox1^+^ VSs, we performed a lineage tracing assay using *tdTomato*^rProx1^ mice, generated by crossing Prox1-CreER^T2^ (ref. ^50^) and *tdTomato* reporter mice. Tamoxifen (50 mg kg^−1^ of body weight) was administered to the pregnant mice at 9.5 days post-coitum, the time of initial LEC specification from cardinal vein ECs based on Prox1 acquisition^51^, and embryos were sampled at E18.5 (Extended Data Fig. 10b). The Prox1-tdTomato signal was not detected in the FOXC2^+^ vascular plexus, but it was distinctly detected in VEGFR3^+^ LVs in the nasal cavity (Extended Data Fig. 10c,d), confirming origination of Prox1^+^ VSs from nascent blood vessels and not from LVs. We validated the lineage tracing assay by confirming that no Prox1-tdT signal was seen in the Cre^−^ mice (Supplementary Fig. 9a,b). The Prox1-tdTomato^+^/E-cadherin^+^/VEGFR3^−^ cells were likely tuft cells in the respiratory epithelium, which highly and selectively express *Lrmp*, *Adgrg6*, *Rgs13* and *Nrgn* (Extended Data Fig. 10c and Supplementary Figs. 2 and 9c). At birth, the Prox1^+^ VSs appeared immature in shape with incomplete lumen formation despite distinctly high Prox1 and FOXC2 in the ECs (Supplementary Fig. 10a). At postnatal day 7 (P7) and P14, the Prox1^+^ VSs were rapidly expanded and matured (Supplementary Figs. 10 and 11) but lacked the typical inward folds, which are seen at P60 (blue arrows in Supplementary Fig. 10b). Thus, Prox1^+^ VSs undergo active maturation processes during postnatal development.

### Hyperplastic expansion of Prox1^+^ VSs with peri-sinusoidal SMC dropout in aged mice

**Fig. 8:**
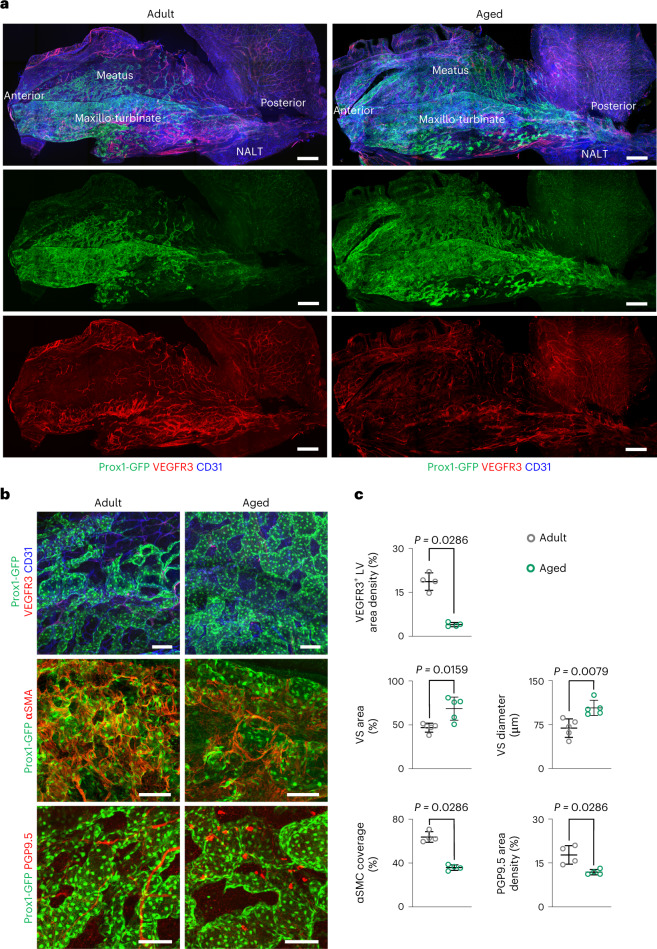
Hypertrophic expansion of Prox1^+^ VSs with peri-sinusoidal SMC dropout in aged mice. **a**, Images of vasculatures in the nasal mucosa of adult (age 3 months) and aged (24–27 months) Prox1-GFP mice. Scale bars, 500 μm. Similar findings are shown from *n* = 4 mice per group from two independent experiments. **b**, Images showing Prox1^+^ VSs, VEGFR3^+^ LVs, αSMA^+^ peri-sinusoidal SMCs and PGP9.5^+^ nerve fibers in nasal mucosa of adult and aged Prox1-GFP reporter mice. Scale bars, 100 μm. Similar findings are shown from *n* = 4–5 mice per group from two independent experiments. **c**, Comparisons of VEGFR3^+^ LV density, area and diameter of Prox1^+^ VSs, αSMA^+^ peri-sinusoidal SMC coverage and PGP9.5^+^ nerve fiber distribution in the nasal mucosa. Each dot indicates a value from one mouse and *n* = 4–5 mice per group from two independent experiments. Data are mean ± s.d*. P* values versus adult by two-tailed Mann–Whitney *U-*test. Source data

Because alterations in vasculature commonly occur with aging^52,53^, we compared changes in nasal mucosal vasculature between adult (age 3 months) and aged (24–27 months) mice. In older compared to younger mice, Prox1^+^ VSs were strikingly expanded up the ceiling of the nasal cavity, and their diameters were increased without apparent change in Prox1 expression, whereas VEGFR3 density and distribution of the LVs were largely reduced (Fig. 8a–c). In parallel, the peri-sinusoidal coverage of the SMCs was apparently compromised, and the densities of SMCs and PGP9.5^+^ nerve fibers were reduced, respectively, by 52% and 22% in aged compared to adult mice (Fig. 8b,c). In the nerve fibers, the density of calcitonin gene-related peptide-positive sensory nerve fibers was reduced by 64%, whereas no change was found in the density of tyrosine-hydroxylase-positive adrenergic nerve fibers in aged compared to adult mice (Supplementary Fig. 12a,b). Therefore, it would be interesting to clarify the cause–effect relationship of those alterations and to see whether the changes lead to a weakened nasal cycle and increased air passage resistance in the nasal turbinate of older subjects.

## Discussion

In this study, we delineated the vascular heterogeneity of the nasal mucosa in mice and humans at the 3D morphological and single-cell molecular levels. Prox1^+^ VSs constitute a major portion of the nasal vasculature, connecting the upstream capillaries and venules to the downstream veins (Supplementary Fig. 6). However, Prox1^+^ VSs surprisingly express the lymphatic-specific transcriptional factors Prox1 and Foxc2. VSs seem responsible for immune surveillance through leukocyte trafficking from the vascular lumen to the submucosa in a VCAM1-dependent manner. LVs also are highly distributed in the nasal mucosa, but the atypically sharp-ended, LYVE1^−^/VEGFR3^+^ lymphatic capillary is common, whereas the typically round-ended, LYVE1^+^/VEGFR3^+^ lymphatic capillary is less common. Both types of lymphatic capillary seem to serve as immune cell drainage. Through scRNA-seq analyses and unsupervised clustering, we identified distinct EC subclusters in the nasal vasculature, which reflect well their distribution and potential functions in the nasal mucosa. Unexpectedly, we found that VSs are regressed or compromised in an AR mouse model and in a peak acute COVID-19 hamster model. Moreover, VSs are expanded and enlarged with SMC loss in aged mice. Thus, our findings clarify 3D morphological and molecular heterogeneities of the nasal vasculature and provide insights into their associations with inflammation, infection and aging in the nasal mucosa.

Each vertebrate tissue and organ has unique blood and lymphatic vasculature that play specific roles and respond to diverse systemic and local stimuli to maintain homeostasis^13,14,16,54^. Earlier and previous studies on nasal vasculature focused on understanding the vascular hierarchy, connections, anastomoses and function in relatively larger animals^1–3,8–11^. Moreover, LVs in the nasal mucosa have been poorly explored, although those in the trachea and lung are well characterized^16,17^. Considerable efforts are underway to decipher, to single-cell resolution, the detailed morphological and molecular microarchitecture of vascular heterogeneity, along with the development of fate maps of the highly heterogeneous vascular cells in most tissues and organs. Nevertheless, deciphering the nasal vascular heterogeneity has been challenging because of its complexity and limited existing knowledge. Therefore, in this study, we used an established microsurgical tissue sampling technique and IFS on whole-mounted tissues from wild-type and Prox1−GFP reporter mice to visualize the nasal vasculature and their surrounding cells at the 3D level, from the embryonic and neonatal periods to the adult and older age periods. This approach facilitated the visualization of a well-organized and exquisite vascular arrangement and complexity in the nasal mucosa. Moreover, our series of scRNA-seq analyses faithfully reflect this morphological and functional pattern and provide unprecedented insight into nasal vascular heterogeneity. Of note, the morphological and molecular landscapes of the nasal vasculature are primarily conserved between mice and humans. Furthermore, the approach of simultaneous i.v. administration of the fluorescence-labeled antibodies allowed us to delineate the vascular hierarchy and connections from the entry to the exit of the mouse nasal blood vasculature. Finding more specific antibodies that can distinguish selectively localized molecules on the luminal surface of ECs in each subtype of the blood vessels would strengthen and expand this application to any tissues and organs.

Nevertheless, this study has limitations, which include: (1) despite using a custom-made tiny mirror, we failed to demonstrate actual blood flow in the mouse nasal vasculature through vital imaging because of the too-small size of the structures; (2) we could not further explore the findings in detail in the COVID-19 model because the analytic instruments were not adequately equipped in our Biosafety Level 3 (BL3) facility; and (3) we have been unable to analyze the human nasal vasculature in detail because obtaining fresh samples during surgery was restricted.

Among the nasal vasculature, VSs constitute a major portion. They are extensively distributed, large-caliber vessels that seem suitable as capacitance vessels for regulating the nasal cycle^1–3,5,6^ (Supplementary Fig. 6). About half of the junctions between ECs of VSs were discontinuous, but, unlike button-like intercellular junctions in initial lymphatics^15,18^, the junctions in the VSs do not have regular spacing with intervening regions without junctional proteins, consistent with distinctive barrier and transport functions of the VSs that remain to be characterized. We unexpectedly found that the ECs composing nasal VSs strongly express two major lymphatic transcriptional factors: Prox1 and Foxc2. In contrast, VSs in the liver and bone marrow do not express either Prox1 or Foxc2 (refs. ^29,37,55^). For this reason, we designated as Prox1^+^ VSs those structures that have been called cavernous sinusoids^9^. Prox1 and Foxc2 play unique, similar or redundant roles in the trans-differentiation of venous ECs into LECs, in lymphangiogenesis and in maintaining the integrity of LVs, including lymphatic valves^34–36,50,54^. In addition, Welsh et al.^38^ recently found that Foxc2 and Prox1 are highly detected in the ECs surrounding the venous valves. They showed that these two transcriptional factors play a crucial role in anti-thrombotic actions, presumably by upregulating anti-thrombotic molecules, such as thrombomodulin, endothelial protein C receptor (EPCR) and tissue factor pathway inhibitor (TFPI), and downregulating the thrombotic proteins vWF, P-selectin and ICAM. Among these molecules, our scRNA-seq analysis showed high levels of EPCR and vWF in the ECs of Prox1^+^ VSs, which may be involved in anti-thrombotic or pro-thrombotic effects on the Prox1^+^ VS ECs that are exposed to a relatively slower blood flow or injury-induced bleeding. Therefore, it is important to explore the detailed roles of Prox1 and Foxc2 in the Prox1^+^ VS using corresponding genetically modified mice.

The nasal mucosa serves as the first physical and immune barrier to constant challenges from inhaled pathogens, but its fragility readily allows for pathogen entry into the submucosa^1–3^. In the submucosa, the invading pathogens are taken up by phagocytic dendritic cells and macrophages, which are presumed to transmigrate into a typical (type 2) lymphatic capillary, a process called ‘passive pathogen entry’, finally draining into the draining LNs and NALT (tonsils in humans) through collecting LVs^13,54,56^. Nevertheless, our findings show the dominant distribution of atypical (type 1) lymphatic capillaries with a zipper-like intercellular junction, which may limit excessive passive pathogen entry into the lymphoid organs. Supporting this idea, a recent report^57^ indicated that the inflammation-induced zippering of lymphatic capillaries serves as a physical barrier to pathogen dissemination by limiting passive pathogen entry. Nevertheless, our findings reveal that type 1 LVs, like type 2 LVs, seem to actively serve as a transmigration conduit for activated immune cells from the nasal mucosa to the draining lymphoid tissues without alteration of the zipper-like intercellular junction under the allergic inflammatory condition. Further investigation on how the immune cells enter and migrate into the type 1 LVs is warranted. Once the pathogen reaches the secondary lymphoid organs, the pathogen-specific CD8^+^ T cells are activated and released into the blood circulation. Eventually, they are recruited to the initial pathogen entry site, such as the nasal mucosa, for immune surveillance, which leads to enhanced mucosal immunity against the pathogen^27^. Given that our Leukocytes^Red^ tracing assay underscores the Prox1^+^ VSs as a primary site of leukocyte adhesion through VCAM1 dependence in both normal and allergic conditions, we regard that they provide a sound platform for trafficking of leukocytes, including the antigen-specific CD8^+^ T cells, from the vascular lumen to the extravascular compartment in the nasal mucosa. Nonetheless, the finding of AR-induced reduction of Prox1^+^ VSs was unexpected but could explain why patients with AR have an impaired nasal cycle and increased airflow resistance through the nasal passage^4^. We regard the markedly increased CD4^+^ cytotoxic T cells and interferon-γ secreted from the Nk2 and ILC-2 cells as the main contributing factors to the observed reductions. Also unexpected were the findings of expanded and enlarged Prox1^+^ VSs accompanied by dropout of peri-sinusoidal SMCs in aged mice. In fact, the phenotypes of vascular aging induced by chronic oxidative stress, low-grade inflammation and mitochondrial dysfunction are diverse^52,53^. Loss of pericyte or perivascular circular SMCs is common with aging in the blood vessels of the brain, retina and kidney^58,59^, leading to abnormal vascular remodeling and function. Likewise, the age-related peri-sinusoidal SMC dropout may cause abnormal expansion of Prox1^+^ VSs, which could attenuate the nasal cycle with age^60^. Indeed, the rhythmicity, reciprocity, frequency and amplitude of the nasal cycle regulated by nasal VSs decline with aging^60^.

Taken together, our findings and the resulting 3D morphologic and molecular atlases highlight the biomedical significance and importance of nasal vasculature. These results can serve as a platform for gaining better insights into nasal cavity homeostasis and pathogenesis of common nasal diseases, such as AR and COVID-19.

## Methods

### Study approval and ethics statement

Experiments on the mice were conducted under the approval (no. KA2021-045) of the Animal Care Committee of the Korea Advanced Institute of Science and Technology (KAIST). Dissected tissues of the human nasal cavity were collected under the approval of the institutional review board (IRB) of Yeungnam University Medical Center (no. YUMC 2021-07-008-002) (Supplementary Table 1). Informed consent was obtained from all participants.

Human specimens were not identified with respect to patient identity. The research protocol with the dissected tissues of the human nasal cavity was approved by the IRB of KAIST (no. KH2022-018). The experiment for the SARS-CoV-2 infection into the hamsters was reviewed and approved by the Animal Ethics Committee of Jeonbuk National University (approval no. JBNU-2020-133).

### Mice

*Prox1*-GFP mice^23^, *Prox1*-CreER^T2^ mice^50^ and *VE-cadherin*-CreER^T2^ mice^61^ were transferred, established and bred in specific pathogen-free (SPF) animal facilities at KAIST. C57BL/6J (B6) and R26-tdTomato mice of both genders were purchased from Jackson Laboratory. *Prox1*-GFP mice from E12.5 to 2–3 month-old adults and 20–25-month-old aged of both genders were used. All mice were bred in our SPF animal facility and fed a standard chow diet (PMI LabDiet) with water. Mice were anesthetized with i.p. injection of a combination of anesthetics (80 mg kg^−1^ ketamine and 12 mg kg^−1^ xylazine) before the experiment or before being sacrificed.

### Sample preparations for histological analyses

Mice were perfused with ice-cold PBS followed by 2% paraformaldehyde (PFA) through the left ventricle by puncturing the right atrium. For whole-mount preparation of the lateral side of the nasal mucosa, the associated skin and muscles were stripped from the face, and then the skull, brain and palates were removed. The nasal cavity was opened by an incision along the midline of the nose while the septum was removed. The dissected tissue containing nasal mucosa and NALT was carefully detached from the inside face skull after the removal of the major portion of the olfactory epithelium. The detached tissue was fixed with 2% PFA for 2 hours at 4 °C. For cross-section preparation of the nasal cavity, the associated skin and muscles were stripped from the face, and then the skull and brain were removed. The whole nose was fixed with 2% PFA for 4 hours at 4 °C, decalcified with 0.5 M EDTA pH 7.4 at 4 °C for 1 day, embedded in 3% low-melting agaroses (Invitrogen) and cut into 200-μm-thick coronal section using a vibrating microtome (VT1200S, Leica). For whole-mount preparation of the nasal cavity of embryos, a midline incision was made on the head, and the nasal cavity was carefully opened. Then, dissected tissue containing the nasal cavity was fixed with 2% PFA for 2 hours at 4 °C, and the attached face skin and connective tissues were removed from the nasal mucosal tissue. The normal portion of dissected tissue containing the inferior turbinate of the human nasal cavity was obtained during the inferior turbinoplasty for patients with the severe deviated nasal septum (Supplementary Table 1). Then, the tissues were fixed with 2% PFA for 2 hours at 4 °C, dehydrated with 30% sucrose in PBS overnight, frozen and embedded in Frozen Section Media (Leica) and cut into 20-μm-thick sections using a Cryocut Microtome (Leica).

### OVA-AR mouse model

To generate an OVA-AR mouse model, the series of i.p and intranasal (i.n.) administrations of OVA were conducted in adult *Prox1*-GFP mice according to a previous report^43^. In brief, the mice were sensitized with triple i.p. administrations of 50 µg of OVA (grade V, Sigma-Aldrich, dissolved in 150 µl of PBS) with 150 µl of Imject Alum (77161, Thermo Fisher Scientific) at a 7-day interval followed by challenge with daily i.n. instillation of 500 µg of OVA dissolved in PBS (30 µl) for 7 days. The mice were treated with the same volume of PBS in the same manner regarded as control. To block the adhesion-molecule-dependent leukocyte trafficking, a cocktail of 200 μg each of rat anti-VLA4 antibody (BE0071, Bio X Cell) and rat anti-LFA1 antibody (BE0006, Bio X Cell) was i.p. injected with every alternative day for four times just after the initial nasal challenge. As a control, a cocktail of the same dose of rat anti-mouse IgG2b isotype (BE0090, Bio X Cell) and rat anti-mouse IgG2a isotype (BE0089) was treated in the same manner. To enrich ECs from the nasal mucosa for the scRNA-seq analysis, the OVA-AR model and its control were separately generated using a *tdTomato*^*rEC*^ mouse as described below.

### Leukocyte recruitment through Prox1^+^ VSs

To determine whether Prox1^+^ VS is a primary site for the recruitment of circulating leukocytes in the nasal vasculature, we first isolated leukocytes from the spleen of B6 adult mice by mincing, rinsing, centrifugation, removal of RBCs by lysis buffer and resuspension according to a previous report^62^. The freshly isolated leukocytes were incubated with CellTracker Red CMTPX Dye (C34552, Thermo Fisher Scientific) according to the manufacturer’s instructions. In total, 1 × 10^7^ of the dye-labeled leukocytes was intravenously injected into the adult Prox1-GFP reporter mice, which were sensitized with the intravenously and repeatedly administered OVA, followed by challenge with single i.n. instillation of 500 µg of OVA dissolved in PBS (30 µl) 1 hour before. At 1 hour after the injection of dye-labeled leukocytes, the mice were perfused with ice-cold PBS followed by 2% PFA through the left ventricle by puncturing the right atrium. To block the adhesion-molecule-dependent leukocyte trafficking, anti-VLA4 antibody (BE0071, Bio X Cell) was i.p. injected 1 day before the i.n. administration of OVA. As a control, the same dose of rat anti-mouse IgG2b isotype (BE0090, Bio X Cell) was treated in the same manner.

### Blood flow analyses in Prox1^+^ VSs

For direct imaging, the nasal mucosa of *Prox1*-GFP mice was rapidly (within 10 minutes) exposed by dissection of surrounding and attached tissues as described above. For lectin or dextran perfusion assay, 150 µl of *Lycopersicon esculentum* agglutinin (LEA, tomato lectin; DL-1177, Vector Labs) or tetramethylrhodamine-labeled dextran (2,000 kDa, lysine fixable; D7139, Invitrogen) was i.v. injected into *Prox1*-GFP mice through the tail vein. At the indicated timepoint later, the mice were sacrificed with the anesthesia, and the nasal mucosa was rapidly exposed. Images were taken in a dark room using a fluorescence stereo zoom microscope (AxioZoom V16, Carl Zeiss).

### IFS

For IFS, the tissues were permeabilized and blocked with blocking buffer containing 5% donkey serum in 1% Triton-X 100 in PBS for 1 hour at room temperature. Then, they were incubated with a primary antibody diluted a ratio of 1:400 in the blocking buffer overnight at 4 °C. The following primary antibodies were used: anti-LYVE1 (rabbit polyclonal, 11-034, AngioBio); anti-CD31 (rat monoclonal, MEC 13.3, 557355, BD Biosciences); anti-CD31 (hamster monoclonal, 2H8, MAB1398Z, Merck); anti-CD31 (sheep polyclonal, AF806, R&D Systems); anti-VE-cadherin (goat polyclonal, AF1002, R&D Systems); anti-VE-cadherin (rat monoclonal, 11D4.1, 550548, BD Biosciences); anti-FOXC2 (sheep polyclonal, AF6989, R&D Systems); anti-FOXC2 (rabbit polyclonal, 23066-1-AP, Proteintech); anti-αSMA-Cy3 (mouse monoclonal, 1A4, C6198, Sigma-Aldrich); anti-VEGFR3 (goat polyclonal, AF743, R&D Systems); anti-VEGFR2 (goat polyclonal, AF644, R&D Systems); anti-endomucin (rat monoclonal, V.5C7, MAB2624, Millipore); anti-GLUT1 (rabbit polyclonal, 07-1401, Millipore); anti-CD3e (hamster monoclonal, 145-2C11, 553058, BD Biosciences); anti-TER119 (rat monoclonal, TER-119, 14-5921-82, eBioscience); anti-PGP9.5 (rabbit monoclonal, D3T2E, 13179, Cell Signaling Technology); anti-MUC5B (mouse monoclonal, 19.4E, ab77995, Abcam); anti-Iba1 (rabbit polyclonal, 019-19741, Wako); anti-MHCII (rat monoclonal, M5/114.15.2, 14-5321-82, eBioscience); anti-B220 (rat monoclonal, RA3-6B2, 553084, BD); anti-PLVAP (rat monoclonal, MECA-32, 550563, BD Biosciences); anti-CCL21 (goat polyclonal, AF457, R&D Systems); anti-PDGFRβ (rat monoclonal, APB5, ab91066, Abcam); anti-vWF (rabbit polyclonal, A0082, Dako); anti-VCAM1 (rat monoclonal, 429(MVCAM.A), 550547, BD Biosciences); anti-ICAM1 (rat monoclonal, YN1/1.7.4, ab119871, Abcam); anti-Tie2 (goat polyclonal, AF762, R&D Systems); anti-podoplanin (mouse monoclonal, D2-40, M3619, Dako); anti-Prox1 (rabbit polyclonal, 102-PA32AG, ReliaTech); anti-CGRP (goat polyclonal, ab36001, Abcam); anti-TH (rabbit polyclonal, AB152, Millipore); anti-claudin5 (rabbit polyclonal, 34-1600, Invitrogen); and anti-ZO1 (rabbit polyclonal, 61-7300, Invitrogen). After several washes with PBS, they were incubated with Alexa Fluor 488-, 594- or 647-conjugated anti-rabbit (711-545-152, 711-585-152, 711-605-152), anti-rat (712-545-153, 712-585-153, 712-605-153), anti-mouse (715-545-151, 715-585-151, 715-605-151), anti-goat (705-545-147, 705-585-147, 705-605-147), anti-sheep (713-545-147, 713-585-147, 713-605-147) and anti-hamster (127-545-160, 127-585-160, 127-605-160) secondary antibodies (Jackson ImmunoResearch) diluted at a ratio of 1:1,000 in the blocking buffer for 4 hours at room temperature. After several washes with PBS, they were mounted with VECTASHIELD (Vector Laboratories). For visualizing the blood vessel continuity according to a previous report^28^, 25–30 µg of fluorescence-labeled antibodies or primary antibodies were i.v. injected into Prox1−GFP mice 15–30 minutes before sacrifice. The following antibodies were used: anti-PODXL (goat polyclonal, AF1556, R&D Systems); anti-PODXL (rat monoclonal, 192703, MAB1556, R&D Systems); anti-Ly6c (rat monoclonal, Monts1, BE0203, Bio X Cell); anti-endomucin (goat polyclonal, AF4666, R&D Systems); anti-PLVAP (rat monoclonal, MECA-32, 550563, BD Biosciences); and BV421-conjugated anti-CD31 (rat monoclonal, MEC 13.3, 562939, BD Biosciences). For fluorescence labeling of the antibody, DyLight conjugation kit (Abcam) was used according to the manufacturer’s instructions. All the antibodies used in this study were validated for the species and applications by the indicated manufacturers. Nuclei were stained with DAPI (Invitrogen) with 1:1,000 dilution. The samples were then mounted with a fluorescent mounting medium (VECTASHIELD).

### Imaging and morphometric analyses

Immunofluorescent images were acquired using an LSM800 or an LSM880 confocal microscope (Carl Zeiss). ZEN 2.3 software (Carl Zeiss) was used for the acquisition and processing of images. Confocal images of whole-mounted samples were maximum intensity projections of tiled or single plane *z*-stack images through the entire thickness of tissues, which were all taken at a resolution of 512 × 512 or 1,024 × 1,024 pixels with the following objectives: air objectives Plan-Apochromat ×10/0.45 numerical aperture (NA) M27 and Plan-Apochromat ×20/0.8 NA M27; LD C-Apochromat ×40/1.1 NA water immersion Corr M27 (LSM 880) with multi-channel scanning in the frame. 3D reconstruction and the cut images were created from *z*-stack confocal images using the 3D tab and the Cut tab in ZEN 2.3 software. Morphometric measurements were performed using ImageJ software (National Institutes of Health) and Zen software. Prox1^+^ VS area was measured in a 500 μm × 500 μm or a 5 mm × 5 mm field in the turbinate and meatus area and presented as a percentage. VS diameter was analyzed in six 500 μm × 500 μm fields per sample. Densities of VEGFR3, PGP9.5, CD3e^+^ T cells, B220^+^ B cells, MHCII^+^ cells, IbaI^+^ cells, TH, CGRP, αSMA^+^ SMC coverage and VCAM1 intensity were presented as a percentage or fold change in a 500 μm × 500 μm field per sample. Vessel densities of VEGFR3^+^/LYVE1^+^ and VEGFR3^+^/LYVE1^−^ were measured in a 5 mm × 5 mm field and presented as a percentage. PLVAP in Prox1^+^ VS and Prox1^−^/VE-cadherin^+^ capillary were first measured in 500 µm × 500 µm field and then normalized to vessel area in a 500 µm × 500 µm field. A number of fenestrations were manually counted in a 800 nm × 800 nm field. The number of CD3e^+^ cells and MHCII^+^ cells in the type 1 LVs and the number of vascular-adherent Leukocytes^Red^ within Prox1^+^ VS sinus were manually counted in a 500 µm × 500 µm field. Number and diameter of human nasal VS and LV were measured in a 3 mm × 3 mm field of the nasal turbinate.

### Transmission electron microscopy

After the transcardial perfusion of adult B6 mice with 4% PFA and 2.5% glutaraldehyde in 0.1 M phosphate buffer (pH 7.4), the respiratory mucosa portion of the nasal cavity was dissected, fixed with 2.5% glutaraldehyde-containing buffer overnight at 4 °C and post-fixed with 1% osmium tetroxide containing buffer for 2 hours at room temperature. Then, the samples were dehydrated with a series of increasing ethanol concentrations followed by resin embedding. Then, 70-nm-thick ultra-thin sections onto copper grids were obtained with an ultramicrotome (Ultracut UCT, Leica). After staining with 2% uranyl acetate and lead citrate, images were taken by a transmission electron microscope (Tecnai G2 Spirit Twin, FEI) at 120 kV.

### Lineage tracing assay for Prox1^+^ VS formation

*tdTomato*^rProx1^ mice were generated by mating *Prox1*-CreER^T2^ mice^50^ and R26-tdTomato mice. *tdTomato*^rProx1^ embryos were generated by timed mating *tdTomato*^rProx1^ male and B6 wild-type female mice, and the day of prominent plug formation in the female mice was regarded as E0.5. Two milligrams of tamoxifen (Sigma-Aldrich) dissolved in corn oil (Sigma-Aldrich) was i.p. injected into the pregnant mice at E9.5, the time of initial LEC specification from the cardinal vein ECs. We sampled and analyzed the tdTomato signal in the nasal mucosa vasculature at E18.5.

### SARS-CoV-2 infection into Syrian hamsters and histopathological examinations

SARS-CoV-2 infection into hamsters was performed according to our previous report^48^. All virus preparations and animal experiments were performed in the BL3 facility and the animal BL3 (ABL3) facility of the Korea Zoonosis Research Institute (KOZRI) at Jeonbuk National University. The researchers who conducted this study were approved and qualified for ABL3 experiments by KOZRI and/or the Bioethics Information Center. SARS-CoV-2 (NCCP43326) was obtained from the Korea Disease Control and Prevention Agency and cultured in African green monkey kidney epithelial cells (Vero E6, CRL-1586, American Type Culture Collection). Male Syrian hamsters (*Mesocricetus auratus*) at 6 weeks of age were obtained from Central Lab Animal (Seoul, South Korea) and maintained in ABL3 conditions under optimal physical environments (24 ± 2 °C, 50 ± 5% humidity). There were six experimental groups according to dpi; 2 dpi, 4 dpi, 8 dpi, 12 dpi, 16 dpi and control (Cont, no infection). After 3 days of adaptation, 1 × 10^6^ plaque-forming units (PFU) of SARS-CoV-2 in a 100-μl volume was inoculated intranasally to each hamster under a light isoflurane anesthesia. On each dpi, infected hamsters were euthanized; the heads and necks were harvested; and they were fixed in 10% neutral buffered formalin and embedded in paraffin. The paraffine-embedded tissue blocks were sectioned at 4 μm in thickness and stained with hematoxylin and eosin. The degree of vasculitis of the nasal turbinate was scored in a range of 0–3 by the severity or proportion: 0, non-to-rare immune cell infiltration into/around VPS; 1, mild immune cell infiltration and vascular enlargement; 2, moderate immune cell infiltration and vascular enlargement; and 3, severe immune cell infiltration and vascular enlargement. Immunohistochemistry (IHC) of SARS-CoV-2 nucleocapsid protein (NP) in the sections was performed according to our previous report^48^. All histopathologic examinations were conducted in a double-blind manner with trained pathologists. To quantify IHC results, ten images were randomly captured from each tissue. Image analysis was performed using analysis TS Auto 5.1 (Olympus). The percentage of IHC-positive area was analyzed in defined magnification field and area (400 magnification field, 0.144 mm^2^).

### Droplet-based scRNA-seq

To enrich ECs from the pooled cells of the mouse nasal mucosal tissue for the scRNA-seq, *tdTomato*^*rEC*^ mice were generated by mating *VE-cadherin*-CreER^T2^ mice^61^ and R26-tdTomato mice. Normal or OVA-AR *tdTomato*^*rEC*^ mice were treated with a single i.p. injection of tamoxifen (2 mg per mouse) 3 days before being sacrificed for the sampling. The nasal mucosal tissue was sampled after the removal of the attached and surrounding tissues as described above. For scRNA-seq of human nasal vasculature, the normal portion of mucosal tissues was obtained during the inferior turbinoplasty for patients with the severe deviated nasal septum (Supplementary Table 1). The dissected tissues were cut into small pieces and incubated with the dissociation buffer containing 2 mg ml^−1^ collagenase II (Worthington), 1 mg ml^−1^ dispase (Gibco) and 0.2 mg ml^−1^ DNase I (Roche) in DMEM at 37 °C for 30 minutes with gentle pipetting up and down every 10 minutes. The samples were then filtered through a 70-µm strainer, and then the supernatant was collected. An equal volume of DMEM containing 10% FBS was added. After centrifugation, cells were resuspended in PBS containing 2% FBS. For human samples, cells were stained with FITC anti-CD45 (304006, BioLegend) and APC anti-CD31 (303115, BioLegend) for labeling CD45^−^CD31^+^ ECs. Then, cell sorting for enrichment of mouse tdTomato^+^ cells or human CD45^−^CD31^+^ cells was performed with FACSAria Fusion (Beckton Dickinson). Dead cells were excluded using DAPI (Sigma-Aldrich) staining, and cell doublets were systematically excluded. Either mouse tdTomato^+^ live cells or human CD45^-^CD31^+^ live cells were suspended in 2% FBS/PBS buffer and processed by 10x Chromium Single Cell 3′ Reagent Kit v3 (10x Genomics) following the manufacturer’s protocol. In brief, cells were mixed with reverse transcription (RT) reagent mix and RT primer and loaded to 10x chips. Cells were separated into gel beads in emulsion (GEMs), where transcripts from individual cells were uniquely barcoded. Barcoded transcripts were directly reverse transcribed and amplified to produce cDNA libraries. Size selection of the initial cDNA libraries was performed using SPRI beads (Beckman Coulter). Then, adaptors were ligated and amplified by sample index polymerase chain reaction (PCR). After another round of double-sided size selection, the quality of the final library was checked by Agilent Bioanalyzer High Sensitivity Chip. The Illumina HiSeq X platform sequenced libraries that passed the final quality control.

### Plate-based scRNA-seq (Smart-seq3)

For plate-based scRNA-seq of the Prox1^+^ ECs, the nasal mucosal tissue of *Prox1*-GFP mice was dissected and incubated with the dissociation buffer following the methods described above. To enrich Prox1^+^ ECs, hematopoietic cells and epithelial cells were depleted using MACS (Miltenyi Biotec) with incubation with anti-CD45 and anti-CD326 microbeads (Miltenyi Biotec) for 15 minutes on ice. Then, cell sorting for enrichment of GFP^+^ cells was performed with FACSAria Fusion (Beckton Dickinson). Dead cells were excluded using DAPI (Sigma-Aldrich) staining, and cell doublets were systematically excluded. The GFP^+^ live single cells were sorted directly to each well in a 96-well plate containing the lysis buffer. After sorting, plates were snap-frozen in liquid nitrogen and stored in a −80 °C deep freezer. Plate-based single-cell libraries were generated following the Smart-seq3 protocol^33^. In brief, cells were lysed, and mRNAs were reverse transcribed. cDNAs were amplified and purified using AMPure XP beads (Beckman Coulter). Purified cDNAs were diluted to 100 pg μl^−1^ and tagmented using a Tn5 tagmentation mix (Illumina). Tagmented products were amplified with custom index primers and pooled into a single tube. After final cleanup using AMPure XP beads, resulting libraries were analyzed by TapeStation for quality control. The Illumina HiSeq X platform sequenced libraries passing the quality control.

### Pre-processing of single-cell data

For droplet-based sequencing, sequenced libraries were demultiplexed and aligned either to the mouse reference genome (mm10) or the human reference genome (GRCh38) by Cell Ranger (version 3.0.2). Sequenced libraries were aligned to the mm10 genome for plate-based sequencing by a STAR aligner. Then, aligned reads were merged to produce a raw read count matrix by the featureCount function in the Subread package. Raw expression matrices were constructed using Read10X function in the ‘R’ package Seurat (version 3.1.1). Before clustering analysis, low-quality cells detected with fewer than 1,000 genes and putative dead cells with high mitochondrial percentage (>10% of reads mapped to mitochondrial genes) were discarded. Cells detected with more than 6,000 genes were also considered as doublets and removed. For gene-based quality control, genes detected in fewer than three cells were removed from the raw expression matrix. After quality control of unwanted cells and genes, expression matrices were normalized column-wise, dividing each gene’s unique molecular identifier (UMI) counts by the sum of UMI counts for a given cell. Then, scale factor 10,000 was multiplied, and log_2_ was transformed to yield log counts per million (CPM) equivalent values.

### Clustering analysis

Clustering and downstream analysis were performed by the ‘R’ package Seurat. First, the top 2,000 most variable genes for each dataset were identified by the FindVariableFeatures function with options: selection.method = ‘vst’. For initial dimensionality reduction, principal component analysis (PCA) was performed. The top 20 principal components were selected for further downstream analysis, such as uniform manifold approximation and projection (UMAP) for two-dimensional visualization and shared nearest neighbor (SNN) graph for neighbor detection and cluster identification using the Louvain algorithm. For the endothelial datasets, after initial clustering, the small number of contaminating non-ECs (for example, CD45^+^ immune cells, PDGFRα^+^ stromal cells and E-cadherin^+^ epithelial cells and others) were removed. The remaining CD31^+^/VE-cadherin^+^ ECs were dimensionality reduced and clustered again as described above. For identification of differentially expressed genes across clusters, the FindMarkers function in Seurat was used with options: test.use = ‘MAST’, min.pct = 0.3 and logfc.threshold = 0.3.

### Integration of datasets

For integrative analysis of datasets showing significant batch effects, integration functions in Seurat were used. In total, 2,000 genes with the highest median variability across datasets were determined by the SelectIntegrationFeatures function. Then, the FindIntegrationAncthors function was used to identify anchors before integration. Finally, datasets were integrated using the IntegrateData function. Using the integrated assay, dimensionality reduction and cluster identification were performed for visualization.

### Reference mapping and annotation of the human nasal EC dataset

To project mouse nasal EC annotation to human nasal ECs, we employed reference mapping implemented in Seurat^63^. In brief, transfer anchors between two datasets were identified using the FindTransferAnchors function with inputs of mouse data as reference, human data as a query and reference reduction as ‘pca’. Then, the predictions of human cell type annotations were produced by the TransferData function by using mouse cell type annotations as a reference.

### Analysis of publicly available datasets

For single-cell analysis of sinusoidal ECs in other tissues, raw expression matrices from the reports describing bone marrow^37^ (Gene Expression Omnibus: GSE108892) and liver^29^ (ArrayExpress: E-MTAB-8077) were acquired. After initial clustering, *Pecam1*^+^/*Cdh5*^+^ ECs were selected and subclustered. After unsupervised clustering of ECs, cell subtype annotations were made according to the expressions of marker genes presented in previous reports^29,37^.

### Statistics

No statistical methods were used to predetermine the sample size. The experiments were randomized, and the investigators were blinded to allocation during experiments and outcome assessment. Values are presented as mean ± s.d. Statistical significance was determined by the two-tailed Mann–Whitney *U-*test versus wild-type. Statistical analyses were performed using GraphPad Prism 8.0 (GraphPad Software). Statistical significance was set at *P* < 0.05.

### Reporting summary

Further information on research design is available in the Nature Portfolio Reporting Summary linked to this article.

### Supplementary information

Supplementary Information Supplementary Figs. 1–12 and Supplementary Table 1

Reporting Summary

Supplementary Video 1. Immunolocalization reveals unique vascular connections in the nasal mucosa. Video showing immunolocalization of the nasal vasculature by simultaneous i.v. injection of fluorescent-labeled anti-Ly6c (for detection of arterioles and capillaries) and anti-endomucin (for detection of capillaries and veins) antibodies to Prox1 reporter mice. Scale bars, 500 μm. Video showing capillaries (deep purple arrows, Ly6c^+^/endmucin^+^), venules (yellow arrow, Ly6c^−^/endmucin^+^) and VSs (Prox1^+^/endomucin^+^/Ly6c^−^) from the beginning to the end. White arrow indicates the site where the venule connects with VS. Scale bars, 100 μm. Similar findings were obtained from *n* = 3 mice per group from two independent experiments.

Supplementary Data Source Data for Supplementary Figs. 10 and 12

### Source data

Source Data Fig. 1. Statistical source data in Fig. 1

Source Data Fig. 2. Statistical source data in Fig. 2

Source Data Fig. 5. Statistical source data in Fig. 5

Source Data Fig. 6. Statistical source data in Fig. 6

Source Data Fig. 7. Statistical source data in Fig. 7

Source Data Fig. 8. Statistical source data in Fig. 8

Source Data Extended Data Fig. 2. Statistical source data in Extended Data Fig. 2

Source Data Extended Data Fig. 3. Statistical source data in Extended Data Fig. 3

Source Data Extended Data Fig. 9. Statistical source data in Extended Data Fig. 9

## Data Availability

The scRNA-seq data of this study are available in the National Center for Biotechnology Information’s Gene Expression Omnibus as a SuperSeries under accession code GSE207086. The SuperSeries consists of human datasets (GSE207083), mouse 10x datasets (GSE207084) and mouse Smart-seq3 datasets (GSE207085). All other data supporting the findings in this study are available within the paper and its Supplementary Information. Source data are provided with this paper.
