# Author Correction: Three-dimensional morphologic and molecular atlases of nasal vasculature

**DOI:** 10.1038/s44161-023-00287-x

**Published:** 2023-05-19

**Authors:** Seon Pyo Hong, Myung Jin Yang, Jung Hyun Bae, Du Ri Choi, Young-Chan Kim, Myeon-Sik Yang, Byungkwan Oh, Kyung Won Kang, Sang-Myeong Lee, Bumseok Kim, Yong-Dae Kim, Ji Hoon Ahn, Gou Young Koh

**Affiliations:** 1 Center for Vascular Research, Institute for Basic Science, Daejeon, Republic of Korea; 2 Graduate School of Medical Science and Engineering, Korea Advanced Institute of Science and Technology (KAIST), Daejeon, Republic of Korea; 3 Laboratory of Veterinary Pathology, College of Veterinary Medicine, Jeonbuk National University, Iksan, Republic of Korea; 4 Division of Biotechnology, College of Environmental and Bioresources, Jeonbuk National University, Iksan, Republic of Korea; 5 Laboratory of Veterinary Virology, College of Veterinary Medicine, Chungbuk National University, Cheongju, Republic of Korea; 6 Department of Otorhinolaryngology-Head and Neck Surgery, College of Medicine, Yeungnam University, Daegu, Republic of Korea; 7 Regional Center for Respiratory Diseases, Yeungnam University Medical Center, Daegu, Republic of Korea

**Keywords:** Cardiovascular biology, Molecular medicine

Correction to: *Nature Cardiovascular Research* 10.1038/s44161-023-00257-3, published online 20 March 2023.

This paper was originally published under standard Springer Nature license (© The Author(s), under exclusive licence to Springer Nature Limited). It is now available as an Open Access paper under a Creative Commons Attribution 4.0 International license, © The Author(s). The error has been corrected in the HTML and PDF versions of the article.

