# Supplementary material for: Three-dimensional morphologic and molecular atlases of nasal vasculature
Source: Nat Cardiovasc Res. 2023 Mar 20;2(5):449–66. doi: 10.1038/s44161-023-00257-3 (PMC11358012; doi:10.1038/s44161-023-00257-3)
Supplement: Supplementary file 1 — Supplementary Figs. 1–12 and Supplementary Table 1 [file 44161_2023_257_MOESM1_ESM.pdf]

nature cardiovascular research

Article

https://doi.org/10.1038/s44161-023-00257-3

Three-dimensional morphologic and molecular atlases of nasal vasculature

In the format provided by the authors and unedited

nature cardiovascular research

Article https://doi.org/10.1038/s44161-023-00257-3

Three-dimensional morphologic and molecular atlases of nasal vasculature

In the format provided by the authors and unedited

Supplementary Information

Supplementary Figures 1-12

1. Distribution of vasculature in the mouse nasal cavity.
2. scRNA-seq analysis of the cells of the *tdTomato*^{rEC} mouse nasal mucosa.
3. The blood vessels having GLUT1^{+} ECs are located in the dorsal meatus and septum regions of the nasal cavity.
4. Heatmap visualizing distinctive expression gene profiles of the indicated EC clusters in the isolated Prox1^{+} EC from the nasal mucosae of Prox1-GFP mice.
5. Sinusoidal ECs in the bone marrow and liver do not express *Prox1* and *Foxc2.*
6. Diagram depicting functional networks and connections of vasculatures in the nasal mucosa.
7. scRNA-seq analysis shows changes in the cell subpopulations of *tdTomato*^{rEC} mouse nasal mucosa in the OVA-induced allergic rhinitis model.
8. Changes of protein levels of vascular growth factor receptors in Prox1^{+} VS and LV in the mouse nasal mucosa during embryonic development.
9. Validation of lineage tracing for emergence of Prox1^{+} cells in the nasal mucosa
10. Maturation of the Prox1^{+} VS during postnatal development in mice.
11. Changes of protein levels of vascular growth factor receptors of Prox1^{+} VS, capillary and LV in the mouse nasal mucosa during postnatal development.
12. Reduced nerve innervation in the nasal mucosa of aged mice.

Supplementary Table 1: Clinical characteristics of human subjects.

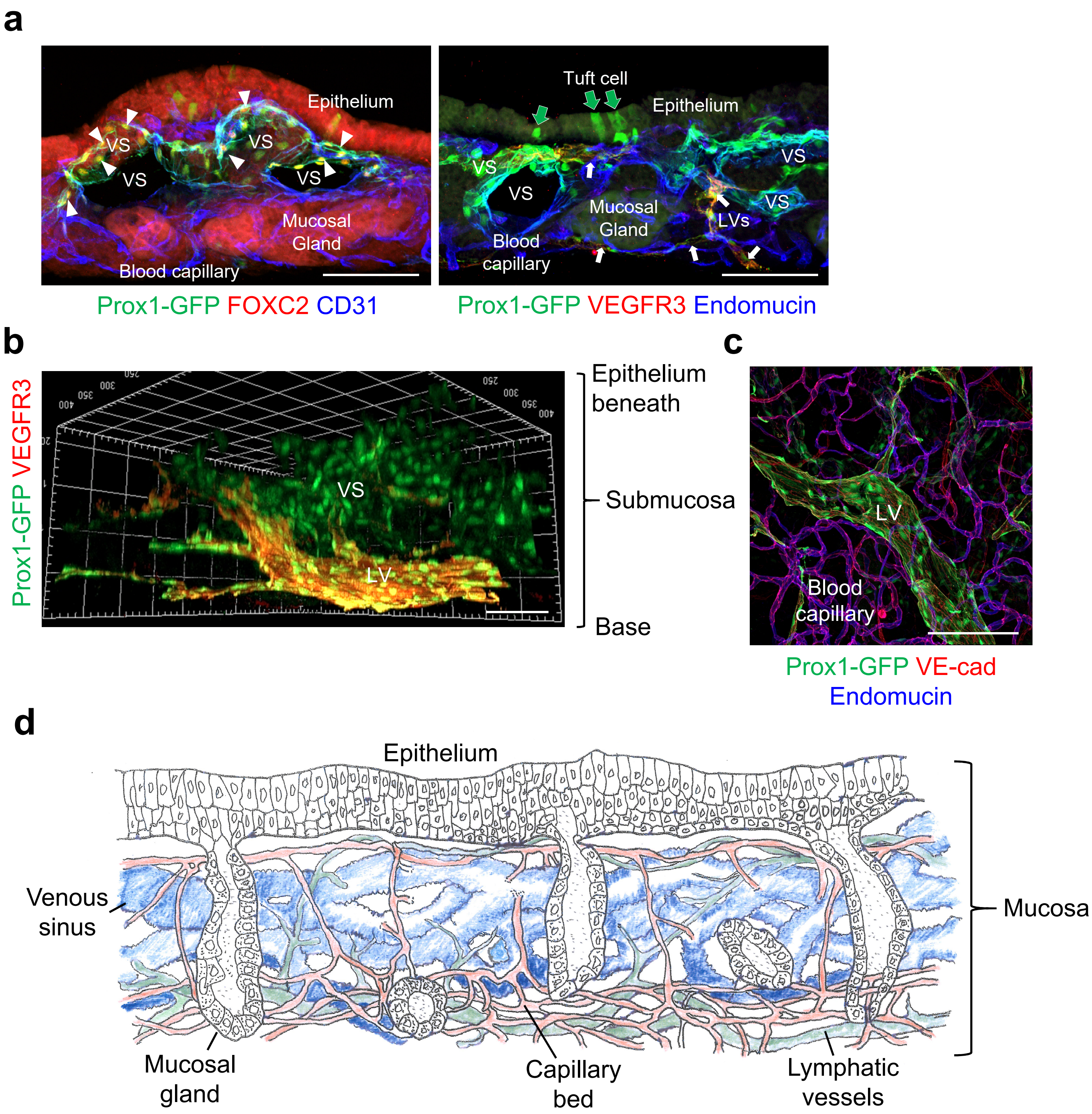

Supplementary Fig. 1. Distribution of vasculature in the mouse nasal cavity

a, Images showing distributions of Prox1⁺/FOXC2⁺ VSs (white arrowheads), Prox1⁺/VEGFR3⁺ LVs (white arrows), Prox1⁻/CD31⁺/endomucin⁺ capillaries in the submucosa and Prox1⁺ tuft cells (green arrows) in the epithelium of cross-sectioned, lateral side nasal mucosa. Scale bars, 100 μm.

b, 3D images showing distributions of Prox1⁺ VSs and Prox1⁺/VEGFR3⁺ LVs in the nasal mucosa. Scale bars, 100 μm.

c, Images showing distributions of Prox1⁺ LVs and endomucin⁺ capillaries in the nasal mucosa. Scale bars, 100 μm.

a-c, Similar findings were obtained from n = 5-6 mice from three to four independent experiments.

d, Diagram depicting distributions of epithelium, thin capillary plexus, VSs, dense capillary plexus, lymphatic network, and mucosal glands in the nasal mucosa.

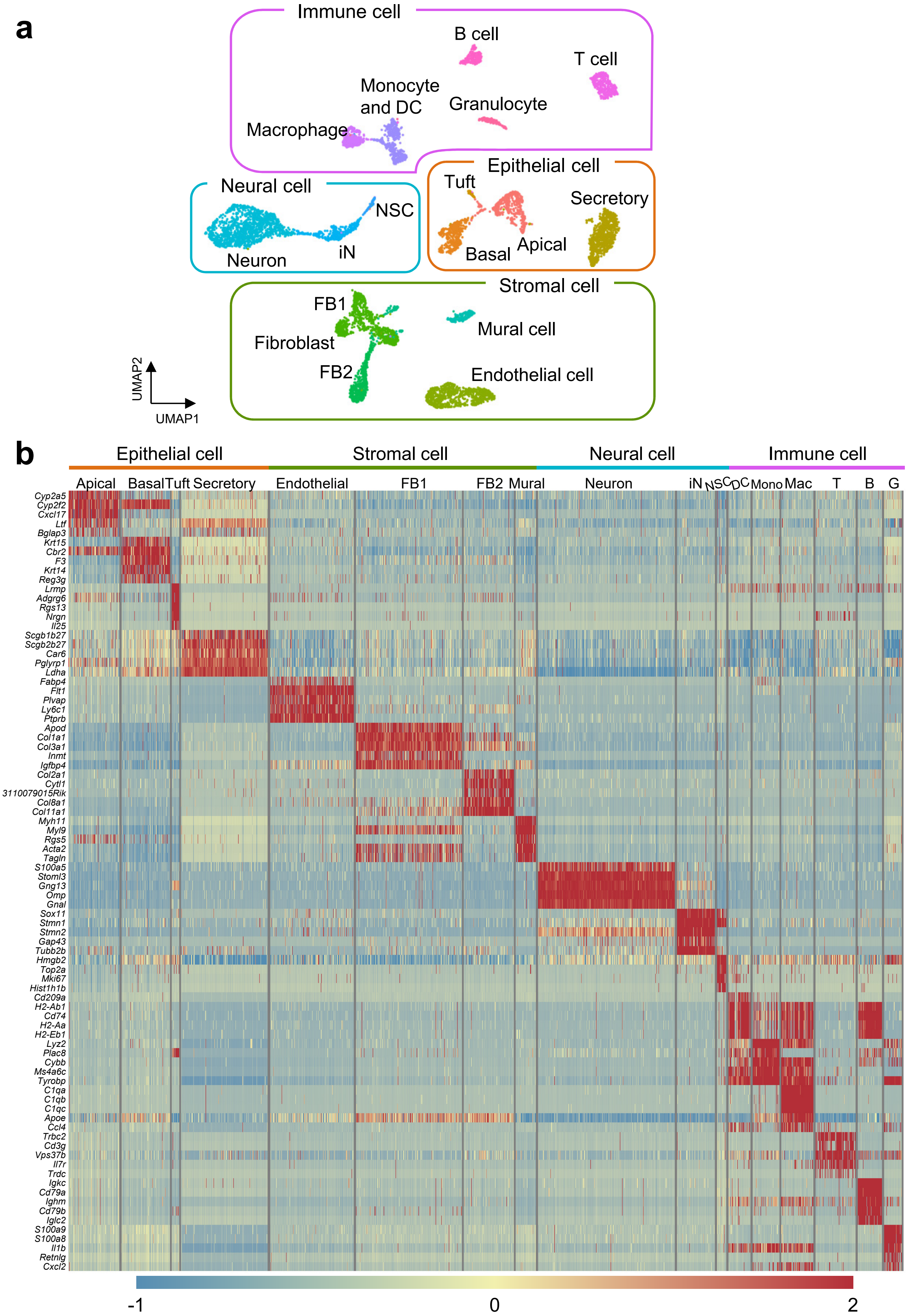

Supplementary Fig. 2. scRNA-seq analysis of the cells of the *tdTomato*^{rEC} mouse nasal mucosa. a, UMAP plots visualizing indicated clusters. **b**, Heatmap visualizing distinctive expression profiles of the indicated cell clusters. Scaled expression levels of top five differentially expressed genes for each indicated cluster are shown.

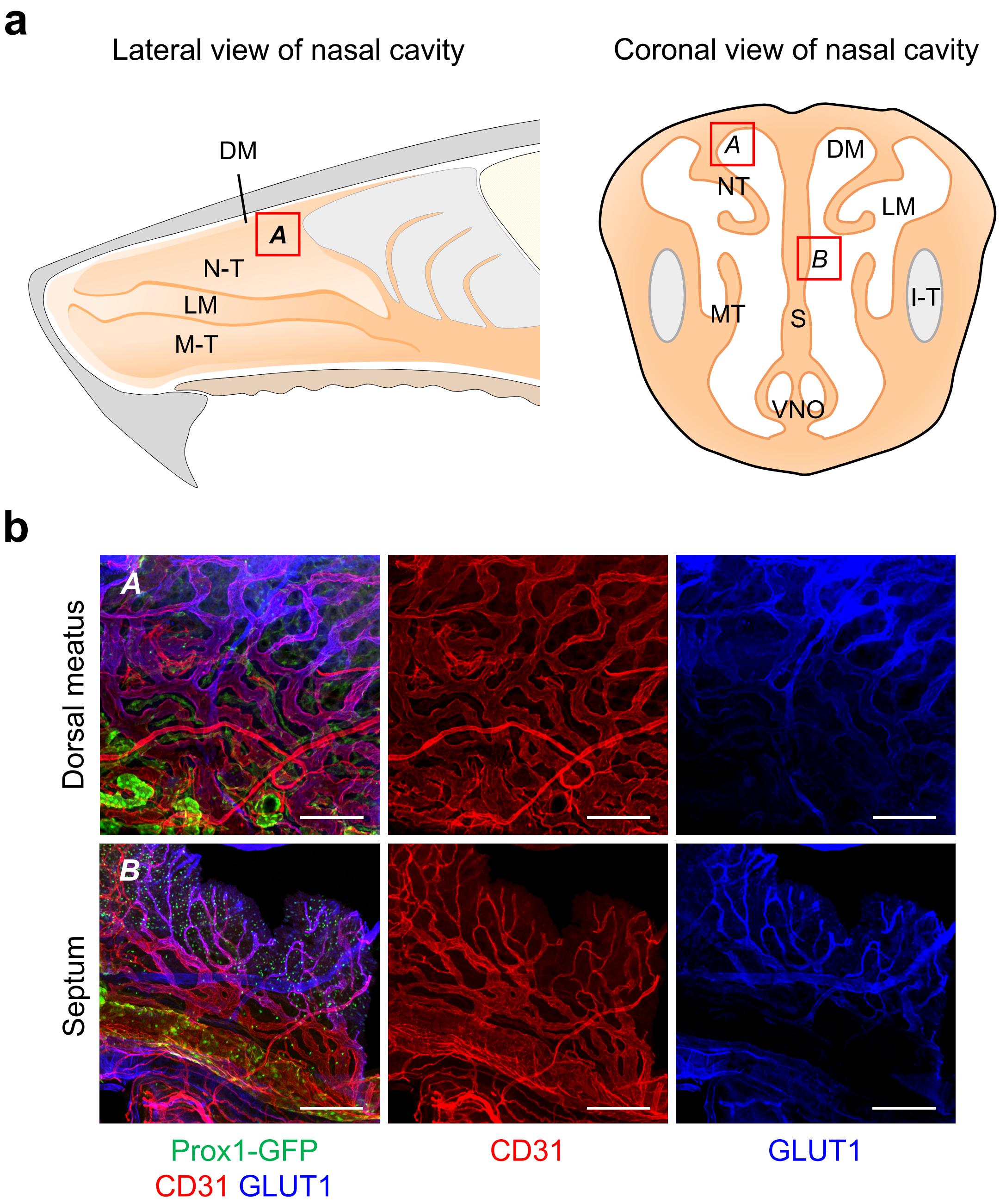

Supplementary Fig. 3. The blood vessels having GLUT1⁺ ECs are located in the dorsal meatus and septum regions of the nasal cavity

a, Illustration of the lateral and coronal view of the mouse nasal cavity. DM, dorsal meatus; LM, lateral meatus; N-T, naso-turbinate; M-T, maxillo-turbinate; I-T, incisor teeth; VNO, vomeronasal organ.

b, Representative images showing GLUT1⁺ blood vessels in the dorsal meatus and septum regions. Scale bars, 200 μm. Similar findings were obtained from n = 3 mice from two independent experiments.

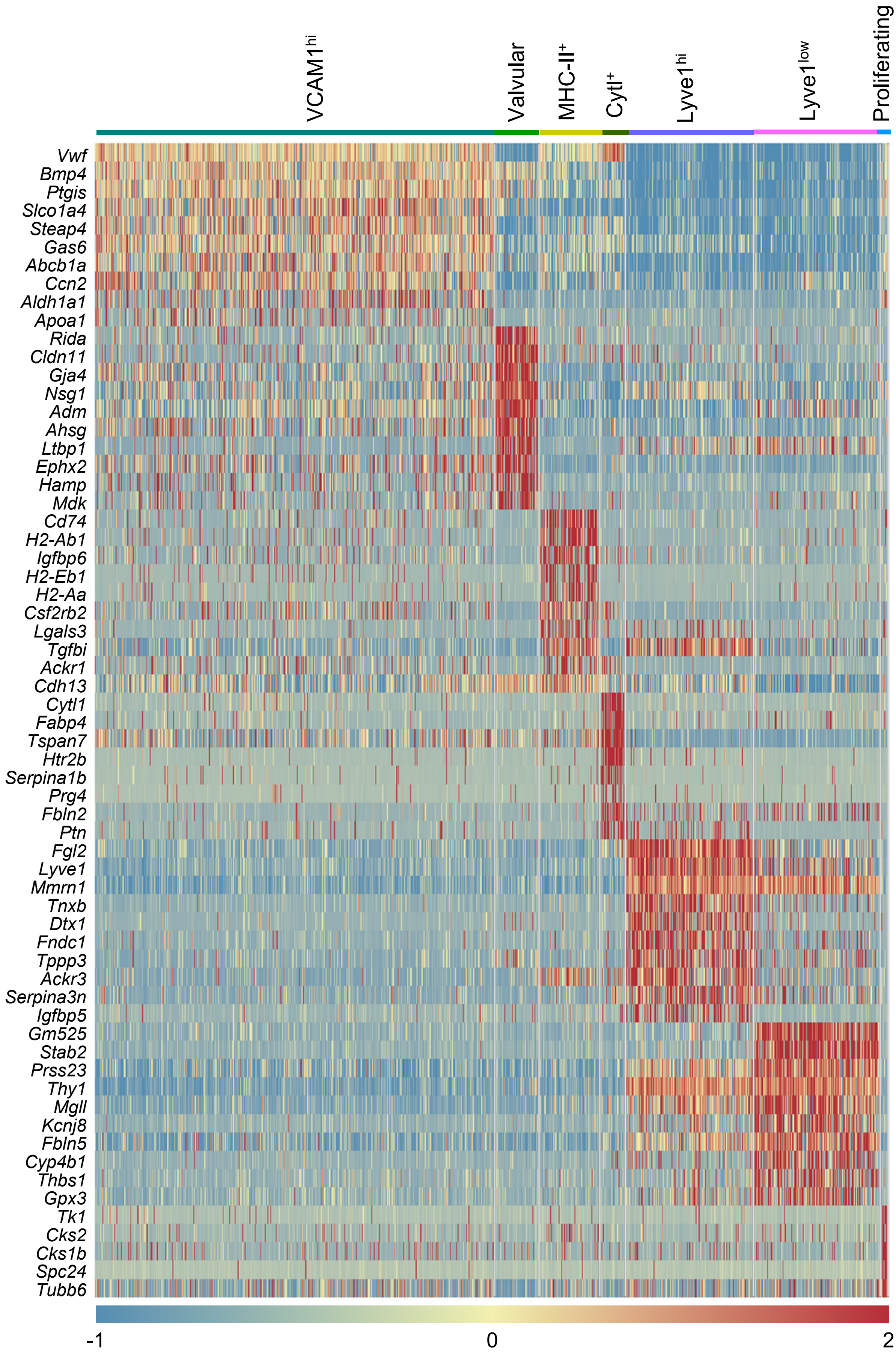

Supplementary Fig. 4. Heatmap visualizing distinctive expression gene profiles of the indicated EC clusters in the isolated Prox1⁺ EC from the nasal mucosae of Prox1-GFP mice. Scaled expression levels of the top 6-10 differentially expressed genes for each indicated cluster are shown.

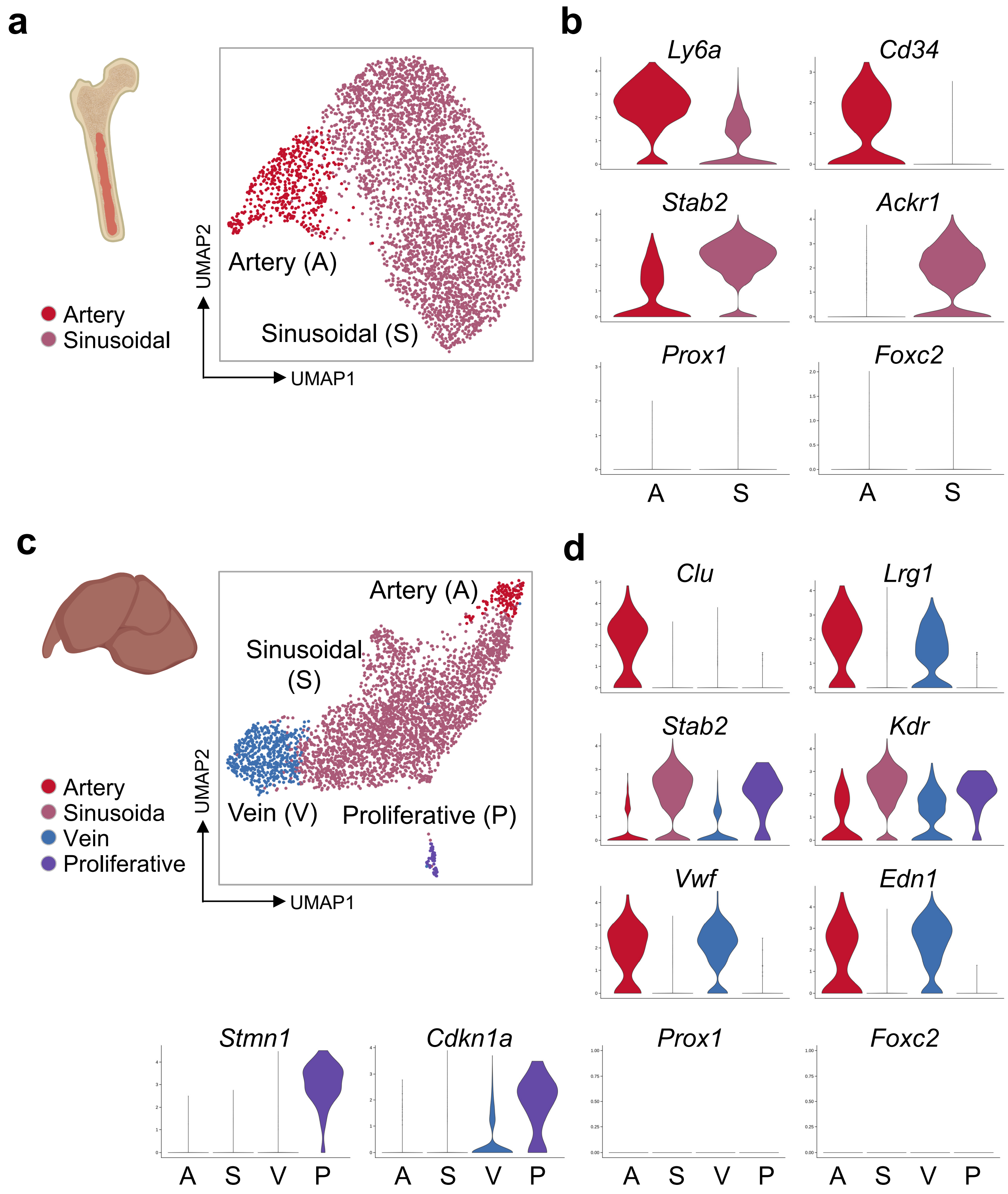

Supplementary Fig. 5. Sinusoidal ECs in the bone marrow and liver do not express *Prox1* and *Foxc2*

a,**b**, UMAP plot showing long bone marrow EC subpopulations- arterial and sinusoidal ECs. Violin plots showing expressions of representative marker genes for artery (*Ly6a* and *Cd34*) and sinusoidal (*Stab2* and *Ackr1*) but no expression of *Prox1* or *Foxc2*.

c,**d**, UMAP plot showing liver EC subpopulations- artery, capillary, vein, and proliferative ECs. Violin plots showing expression of representative marker genes for artery (*Clu* and *Lrg1*), sinusoidal (*Stab2* and *Kdr*), vein (*Vwf* and *Edn1*), proliferative EC (*Stmn1* and *Cdkn1a*) but no expression of *Prox1* or *Foxc2*.

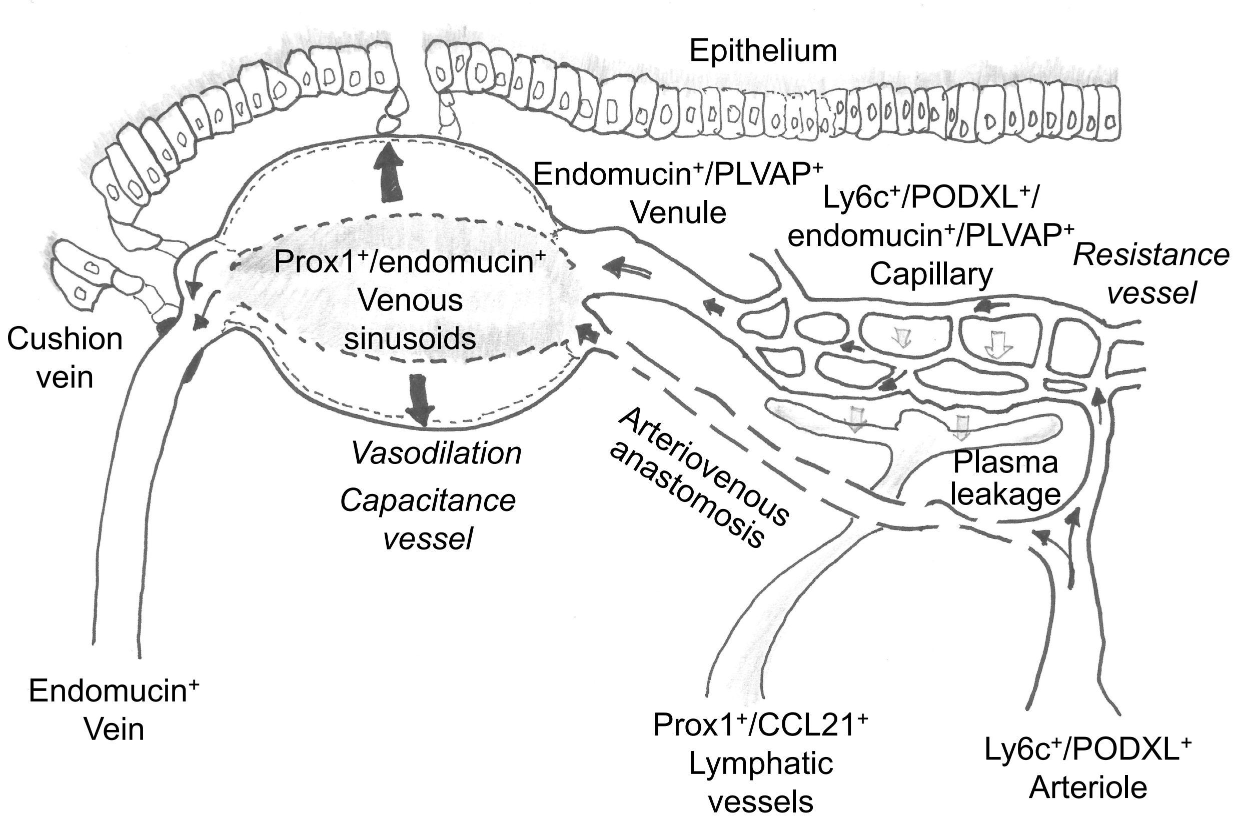

Supplementary Fig. 6. Diagram depicting functional networks and connections of vasculatures in the nasal mucosa. Here, $Prox1^+$ VSs constitute a major portion as large-caliber capacitance vessels for regulating the nasal cycle, while small arteries, arterioles, and arteriovenous anastomoses serve as the resistance vessels for regulating blood flow. No discernable arteriovenous anastomosis (dotted lines) is detected in the mouse nasal mucosa in this study. Lymphatic vessels serve as a drainage route for excessive interstitial fluid and immune cells bearing pathogens invaded by inhaled air (modified the image by Ichimura K., Mechanism of nasal obstruction in patients with allergic rhinitis. *Clinical and Experimental Allergy Reviews* 10:20-27, 2010).

Supplementary Fig. 7

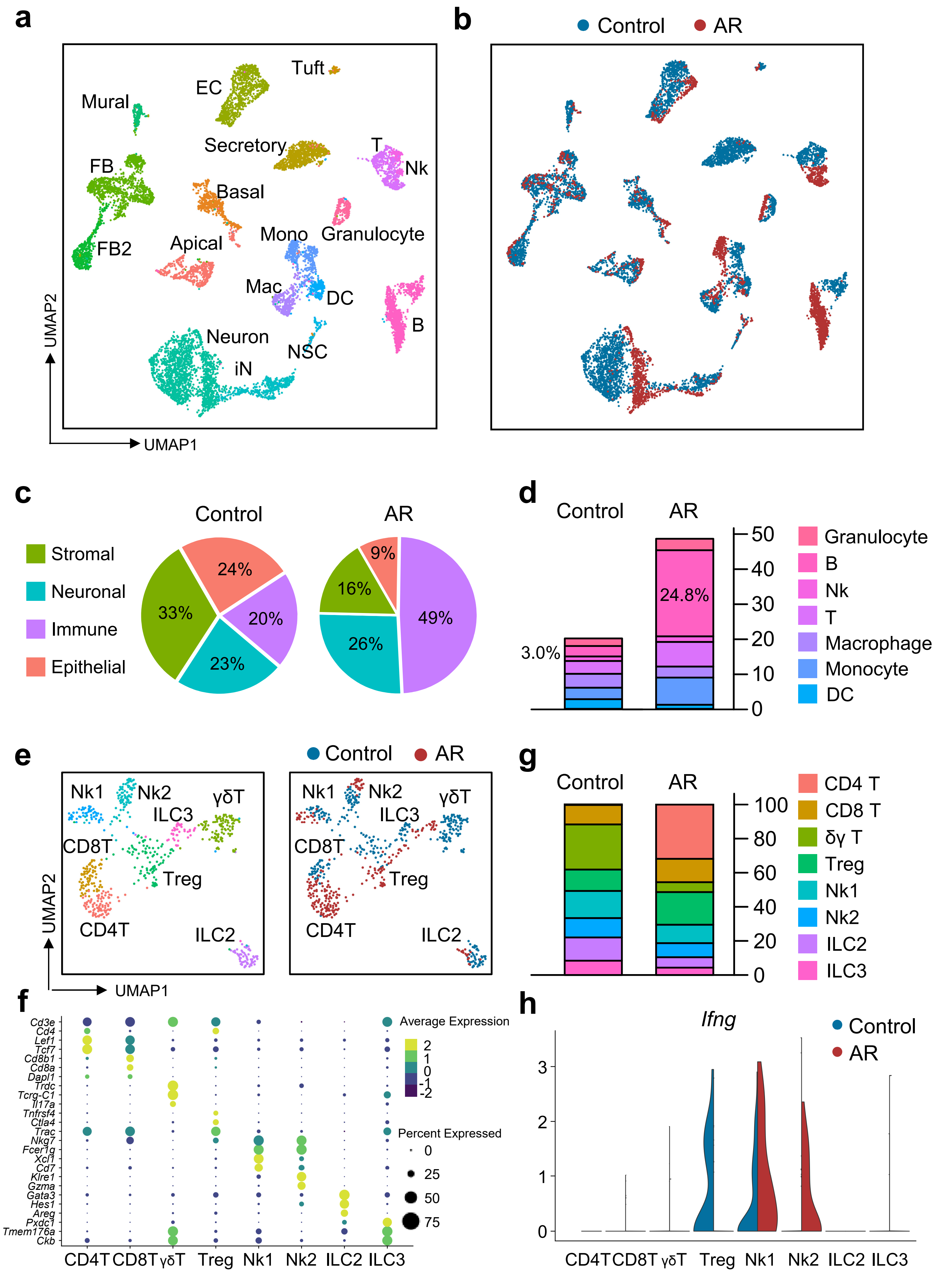

Supplementary Fig. 7. scRNA-seq analysis shows changes in the cell subpopulations of *tdTomato*^{rEC} mouse nasal mucosa in the OVA-induced allergic rhinitis model

a,**b**, UMAP plots visualizing the subpopulations constituting the pooled cells derived from the respiratory mucosa of Control and OVA-AR (AR). FB, fibroblasts; EC, endothelial cells; Nk, natural killer cells; Mono, monocytes; Mac, macrophages; DC, dendritic cells; iN, interneurons; NSC, neuronal stem cells.
c, Pie charts showing different fractions of the indicated main cell populations in the mucosa between Control and AR.
d, Bar plots showing different fractions of the indicated immune cell subpopulations in the mucosa between Control and AR.
e,**f**, UMAP plots depicting the subpopulations constituted the pooled T cells derived from the mucosa of Control and AR. Dot plot visualizing average expression levels of the marker genes that distinguish each T cell subpopulation. Dot size represents the percentage of cells expressing the indicated gene in the indicated cluster.
g, Bar plots showing different fractions of the indicated T cell subpopulations in the mucosa between Control and AR.
h, Violin plot depicting expression changes of *Ifng* between Control and AR in the indicated clusters.

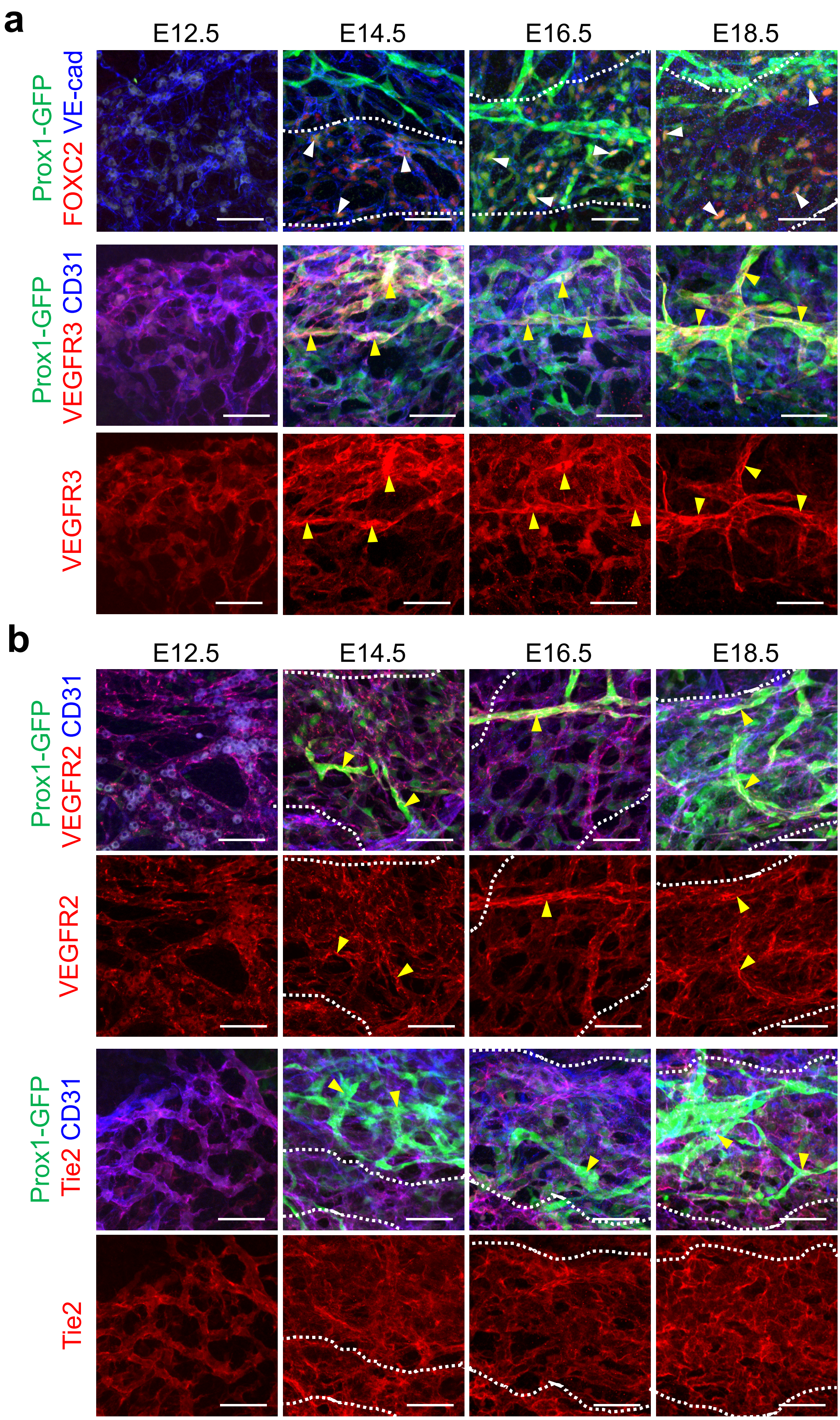

Supplementary Fig. 8. Changes of protein levels of vascular growth factor receptors in Prox1⁺ VS and LV in the mouse nasal mucosa during embryonic development

a,**b**, Images showing Prox1⁺FOXC2⁺ VSs (white arrowheads) and Prox1⁺VEGFR3⁺ LVs (yellow arrowheads) at indicated embryonic ages. Scale bars, 50 μm. Similar findings were shown from n = 4 mice/group from two independent experiments.

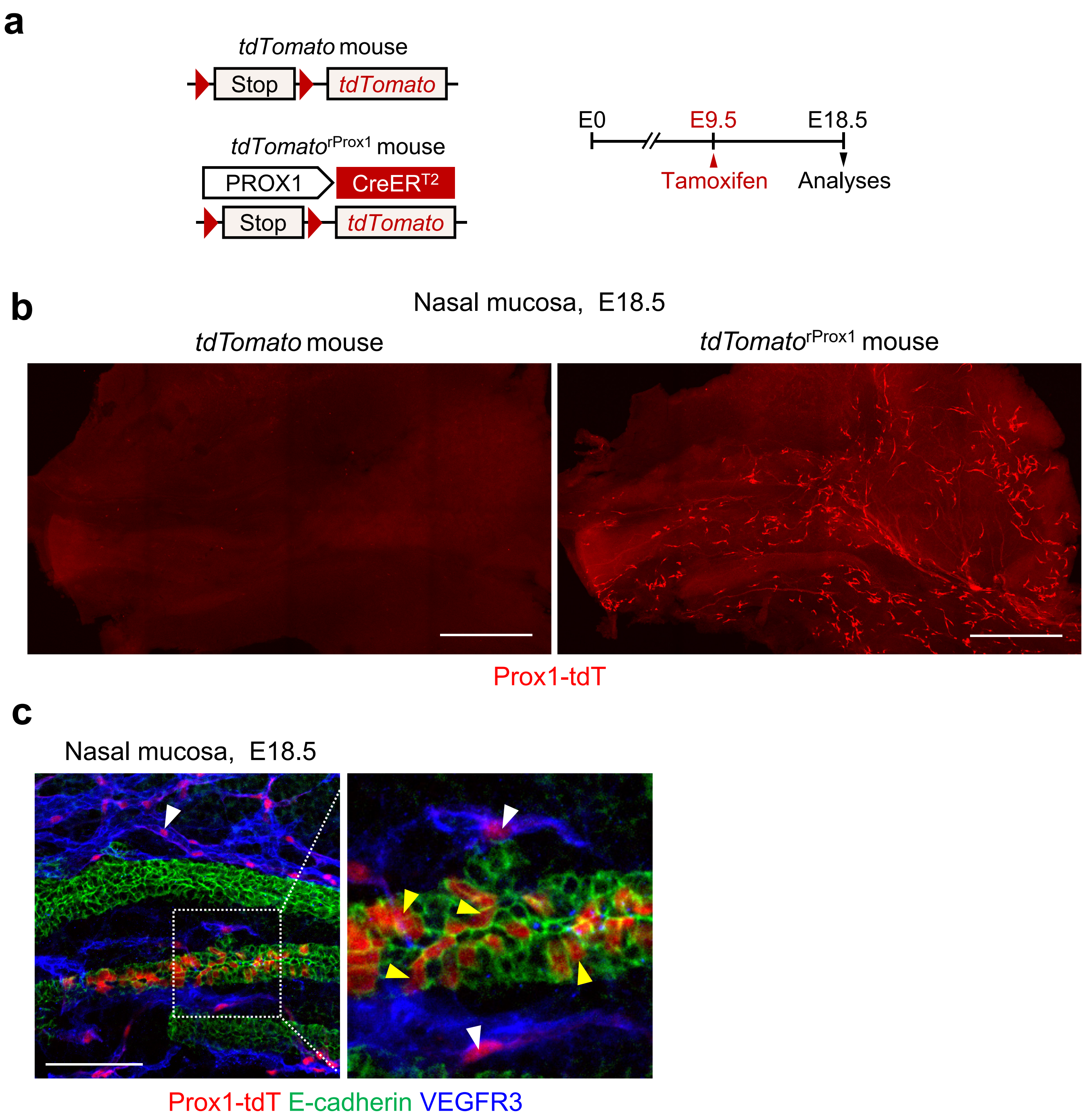

Supplementary Fig. 9. Validation of lineage tracing for emergence of Prox1^{+} cells in the nasal mucosa

a, Diagram depicting generation of *tdTomato*^{rProx1} mouse by crossing Prox1-CreER^{T2} and *tdTomato*-reporter mice for the lineage tracing assay in the nasal mucosa. Tamoxifen administration at E9.5 and sampling at E18.5.

b, Note that clear Prox1-tdTomato (tdT) signals are seen in *tdTomato*^{rProx1} mouse but no Prox1-tdT signal is seen in *tdTomato* mouse. Scale bars, 500 µm. Similar results were obtained from three independent experiments.

c, Representative image showing Prox1-tdTomato (tdT) signal in E-cadherin^{+} epithelial cells (yellow arrowheads) and VEGFR3^{+} lymphatic vessels (white arrowheads) in the nasal mucosa at E18.5 of *tdTomato*^{rProx1} mouse. Scale bar, 100 µm. Note that Prox1-tdT^{+}/E-cadherin^{+} epithelial cells are likely tuft cells. Similar finding was shown from n = 3 mice from two independent experiments.

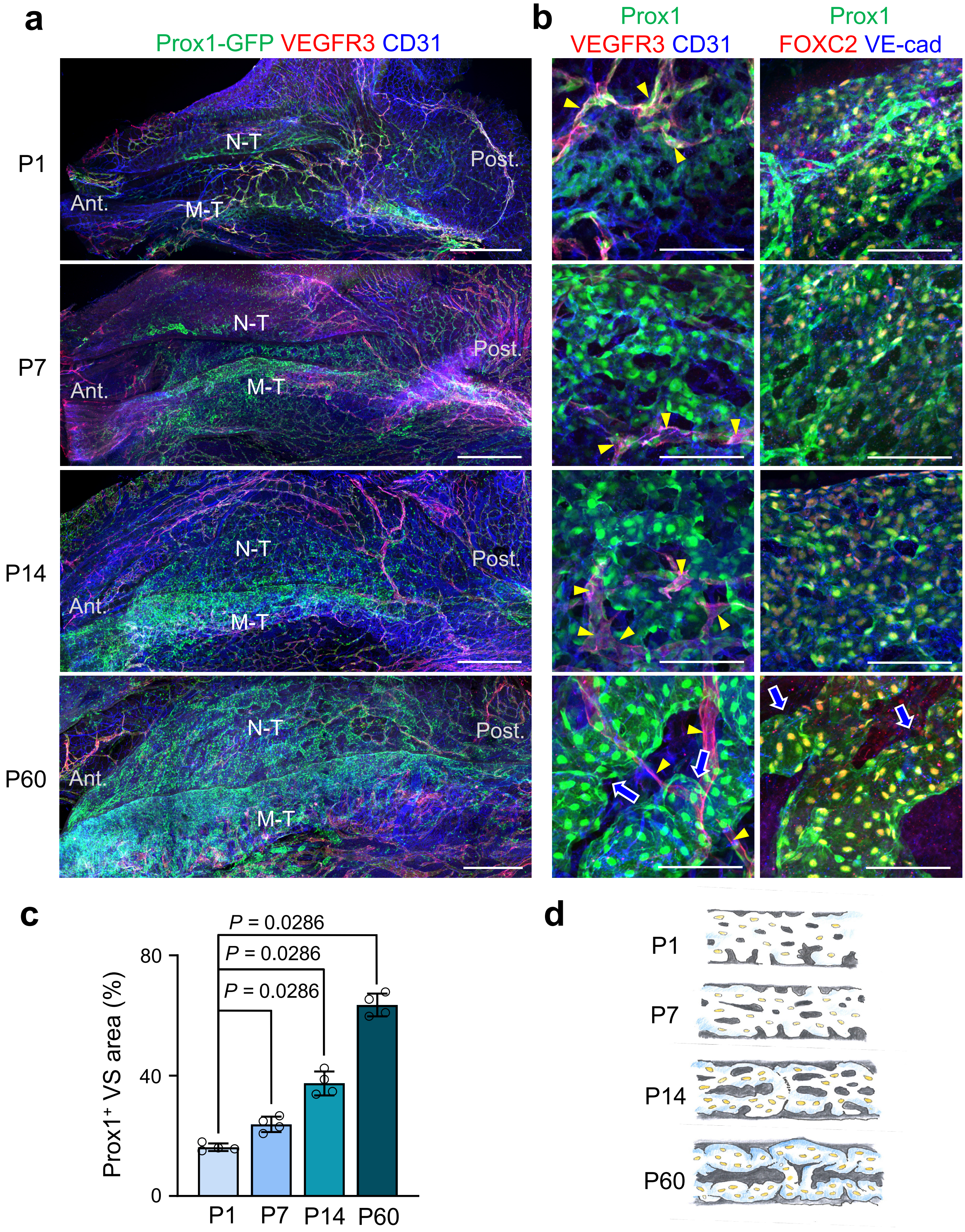

Supplementary Fig. 10. Maturation of the Prox1⁺ VS during postnatal development in mice
a,**b**, Images of Prox1⁺/FOXC2⁺ VSs and Prox1⁺/VEGFR3⁺ LVs (yellow arrowheads) in the nasal mucosa of Prox1-GFP reporter mice at postnatal day 1 (P1), P7, P14 and P60. Note that inward folds (blue arrows) in the Prox1 VSs are seen only P60. N-T, nasoturbinate; M-T, maxilloturbinate. Scale bars, 500 μm (**a**) and 100 μm (**b**). Similar finding was shown from n = 4 mice/group from two independent experiments. **c**, Comparisons of relative Prox1⁺ VS area per nasal mucosa area (%) at the indicated postnatal days. Each dot indicates a value from one mouse and n = 4 mice/group from two independent experiments. Vertical bars indicate mean ± SD. *P* values versus P1 by two-tailed Mann-Whitney *U* test. **d**, Diagram depicting maturation of Prox1⁺ VS during postnatal development.

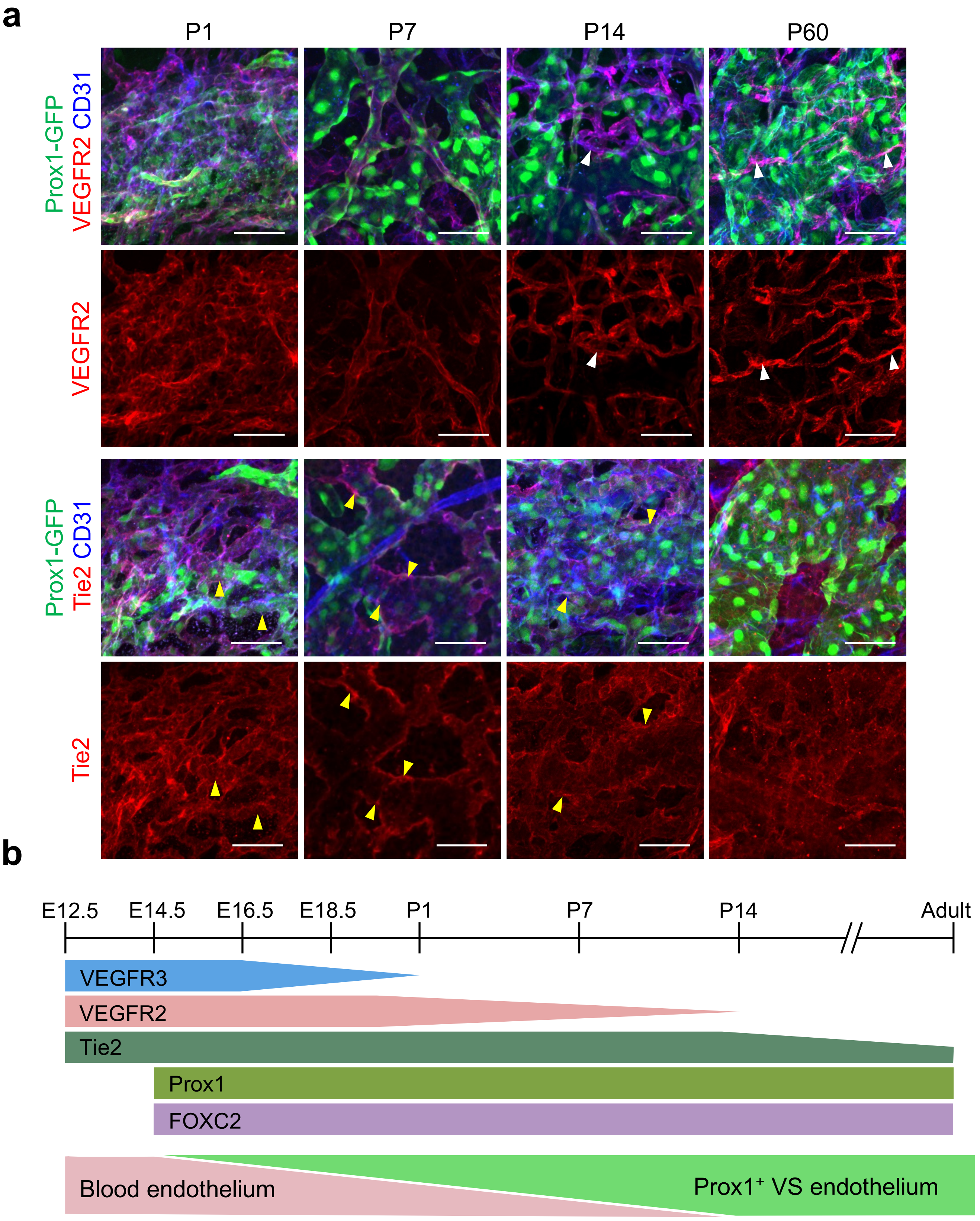

Supplemenatry Fig. 11. Changes of protein levels of vascular growth factor receptors of Prox1⁺ VS, capillary and LV in the mouse nasal mucosa during postnatal development

a, Images showing VEGFR2^{high} capillary (white arrowheads) and Tie2^{high} Prox1^{+} VSs (yellow arrowheads) at P1, P7, P14, and P60. Scale bars, 50 μm. Similar findings were shown from n = 4 mice/group from two independent experiments.

b, Diagram depicting temporal changes of indicated growth factors and transcriptional factors in the Prox1^{+} VSs during postnatal development.

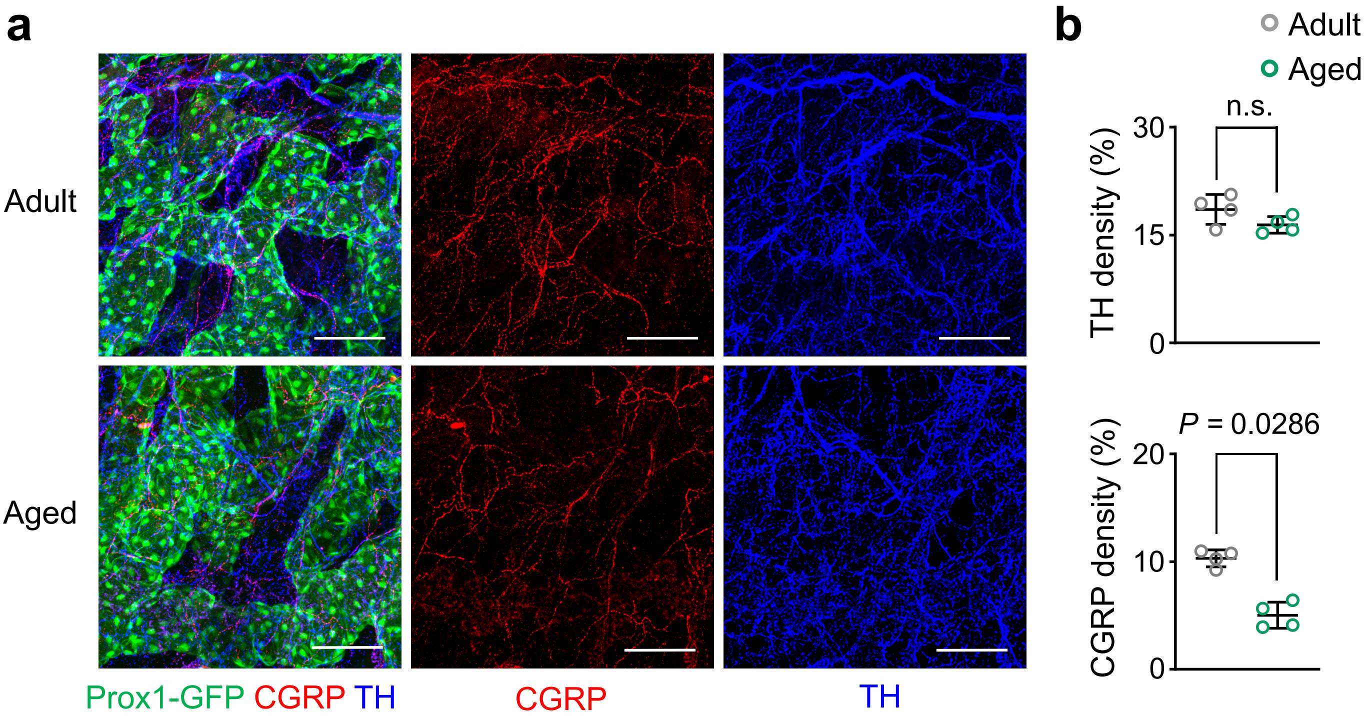

Supplementary Fig. 12. Reduced nerve innervation in the nasal mucosa of aged mice
a,**b**, Images and comparisons of distributions of calcitonin gene-related peptide (CGRP)$^+$ sensory nerve fibers and tyrosine hydroxylase (TH)$^+$ adrenergic nerve fibers in the nasal mucosa of adult (age 3 months) and aged (24-27 months) Prox1-GFP mice. Scale bars, 100 µm. Each dot indicates a value from one mouse and n = 4 mice/group from two independent experiments. Bars indicate mean ± SD. *P* values versus Adult by two-tailed Mann-Whitney *U* test.

Supplementary Table 1. Clinical characteristics of human subjects.

	**Pt1**	**Pt2**	**Pt3**	**Pt4**
Age	19	69	21	38
Sex	F	M	M	F
Disease	Deviated nasal septum	Deviated nasal septum	Deviated nasal septum	Deviated nasal septum
Surgery	Septoplasty with inferior turbinoplasty	Septoplasty with inferior turbinoplasty	Septoplasty with inferior turbinoplasty	Septoplasty with inferior turbinoplasty
