# Supplementary material for: Three-dimensional morphologic and molecular atlases of nasal vasculature
Source: Nat Cardiovasc Res. 2023 Mar 20;2(5):449–66. doi: 10.1038/s44161-023-00257-3 (PMC11358012; doi:10.1038/s44161-023-00257-3)
Supplement: Supplementary file 2 — Reporting Summary [file 44161_2023_257_MOESM2_ESM.pdf]

nature portfolio

Corresponding author(s): Gou Young Koh/Ji Hoon Ahn

Last updated by author(s): Feb 20, 2023

Reporting Summary

Nature Portfolio wishes to improve the reproducibility of the work that we publish. This form provides structure for consistency and transparency in reporting. For further information on Nature Portfolio policies, see our Editorial Policies and the Editorial Policy Checklist.

Statistics

For all statistical analyses, confirm that the following items are present in the figure legend, table legend, main text, or Methods section.

n/a	Confirmed	
☐	☒	The exact sample size (*n*) for each experimental group/condition, given as a discrete number and unit of measurement
☐	☒	A statement on whether measurements were taken from distinct samples or whether the same sample was measured repeatedly
☐	☒	The statistical test(s) used AND whether they are one- or two-sided<br>*Only common tests should be described solely by name; describe more complex techniques in the Methods section.*
☐	☒	A description of all covariates tested
☐	☒	A description of any assumptions or corrections, such as tests of normality and adjustment for multiple comparisons
☐	☒	A full description of the statistical parameters including central tendency (e.g. means) or other basic estimates (e.g. regression coefficient) AND variation (e.g. standard deviation) or associated estimates of uncertainty (e.g. confidence intervals)
☐	☒	For null hypothesis testing, the test statistic (e.g. *F*, *t*, *r*) with confidence intervals, effect sizes, degrees of freedom and *P* value noted<br>*Give P values as exact values whenever suitable.*
☒	☐	For Bayesian analysis, information on the choice of priors and Markov chain Monte Carlo settings
☒	☐	For hierarchical and complex designs, identification of the appropriate level for tests and full reporting of outcomes
☒	☐	Estimates of effect sizes (e.g. Cohen's *d*, Pearson's *r*), indicating how they were calculated

Our web collection on statistics for biologists contains articles on many of the points above.

Software and code

Policy information about availability of computer code

Data collection	LSM image software (Carl Zeiss)<br>Zen 2.3 software (Carl Zeiss)
Data analysis	Zen 2.3 software (Carl Zeiss)<br>GraphPad Prism 8.0 (GraphPad Software)<br>ImageJ (Fiji version) software (NIH)<br>STAR (version 2.6.1d)<br>featureCounts (version 2.0.1)<br>Cell Ranger 3.0.2 toolkit from 10X Genomics (https://10xgenomics.com)<br>R: The R Project for Statistical Computing<br>R: package 'Seurat' (version 3.1.1)

For manuscripts utilizing custom algorithms or software that are central to the research but not yet described in published literature, software must be made available to editors and reviewers. We strongly encourage code deposition in a community repository (e.g. GitHub). See the Nature Portfolio guidelines for submitting code & software for further information.

Data

Policy information about availability of data

All manuscripts must include a data availability statement. This statement should provide the following information, where applicable:

- Accession codes, unique identifiers, or web links for publicly available datasets
- A description of any restrictions on data availability
- For clinical datasets or third party data, please ensure that the statement adheres to our policy

The scRNA-seq data of this study are available in the NCBI's Gene Expression Omnibus as a SuperSeries under accession code GSE207086. The SuperSeries consist of human datasets (GSE207083), mouse 10X datasets (GSE207084), and mouse Smart-Seq3 datasets (GSE207085). All other data supporting the findings in this study are available within the paper and its Supplementary Information. Source data are provided with this paper.

Human research participants

Policy information about studies involving human research participants and Sex and Gender in Research.

Reporting on sex and gender	We obtained the normal portion of dissected tissue containing the inferior turbinate from two male and two female.
Population characteristics	participant 1 : Female, 19 (age), Deviated nasal septum (Disease), Septoplasty with inferior turbinoplasty (Surgery)<br>participant 2 : Male, 69 (age), Deviated nasal septum (Disease), Septoplasty with inferior turbinoplasty (Surgery)<br>participant 3 : Male, 21 (age), Deviated nasal septum (Disease), Septoplasty with inferior turbinoplasty (Surgery)<br>participant 4 : Female, 38 (age), Deviated nasal septum (Disease), Septoplasty with inferior turbinoplasty (Surgery)
Recruitment	We explained our purpose of this study to the patients with the deviated nasal septum and obtained the agreement from the participants with the written informed consent before the sampling under approval of the IRB. We collected the samples upon availability without any self-selection bias or other biases.
Ethics oversight	The human nasal samples were collected under the approval from the IRB of Yeungnam University Medical Center (No. YUMC 2021-07-008-002) in Daegu, Korea. The research of human nasal sample analyses were approved under IRB of KAIST (No. KH2022-018).

Note that full information on the approval of the study protocol must also be provided in the manuscript.

Field-specific reporting

Please select the one below that is the best fit for your research. If you are not sure, read the appropriate sections before making your selection.

☒ Life sciences ☐ Behavioural & social sciences ☐ Ecological, evolutionary & environmental sciences

For a reference copy of the document with all sections, see nature.com/documents/nr-reporting-summary-flat.pdf

Life sciences study design

All studies must disclose on these points even when the disclosure is negative.

Sample size	Sample sizes were chosen on the basis of standard power calculations (with α = 0.05 and power of 0.8) performed for similar experiments and statistical methods were not used to predetermine sample sizes as previously published (Robciuc et al., Cell Metabolism, 2016; Louveau et al., Nature, 2015).
Data exclusions	No samples were excluded from the analysis.
Replication	Experiments were replicated at least once for all analyses to produce convincing results and the number of reproductions of each experimental finding is described in each figure legends. All attempts at experimental replication were successful.
Randomization	Animals were taken from different cages and litters, and randomly allocated based on the experiments.
Blinding	The investigators were blinded during the experiments and the result analyses.

Reporting for specific materials, systems and methods

We require information from authors about some types of materials, experimental systems and methods used in many studies. Here, indicate whether each material, system or method listed is relevant to your study. If you are not sure if a list item applies to your research, read the appropriate section before selecting a response.

Materials & experimental systems

n/a	Involved in the study
☐	☒ Antibodies
☐	☒ Eukaryotic cell lines
☒	☐ Palaeontology and archaeology
☐	☒ Animals and other organisms
☒	☐ Clinical data
☒	☐ Dual use research of concern

Methods

n/a	Involved in the study
☒	☐ ChIP-seq
☒	☐ Flow cytometry
☒	☐ MRI-based neuroimaging

Antibodies

Antibodies used

The following primary antibodies were used: anti-LYVE–1 (rabbit polyclonal, 11-034, Angiobio, 1:400); anti-CD31 (rat monoclonal, MEC 13.3, 557355, BD Biosciences, 1:400); anti-CD31 (hamster monoclonal, 2H8, MAB1398Z, Merck, 1:400); anti-CD31 (sheep polyclonal, AF806, R&D, 1:400); anti-VE–cadherin (goat polyclonal, AF1002, R&D, 1:400); anti-VE-cadherin (rat monoclonal, 11D4.1, 550548, BD Biosciences, 1:400); anti-FOXC2 (sheep polyclonal, AF6989, R&D, 1:400); anti-FOXC2 (rabbit polyclonal, 23066-1-AP, Proteintech, 1:400); anti-αSMA–Cy3 (mouse monoclonal, 1A4, C6198, Sigma-Aldrich, 1:400); anti-VEGFR3 (goat polyclonal, AF743, R&D, 1:400); anti-VEGFR2 (goat polyclonal, AF644, R&D, 1:400); anti-Endomucin (rat monoclonal, V.5C7, MAB2624, Millipore, 1:400); anti-GLUT1 (rabbit polyclonal, 07-1401, Millipore, 1:400); anti-CD3e (hamster monoclonal, 145-2C11, 553058, BD Biosciences, 1:400); anti-TER119 (rat monoclonal, TER-119, 14-5921-82, eBioscience, 1:400); anti-PGP9.5 (rabbit monoclonal, D3T2E, 13179, Cell Signaling, 1:400); anti-MUC5B (mouse monoclonal, 19.4E, ab77995, Abcam, 1:400); anti-Iba1 (rabbit polyclonal, 019-19741, Wako, 1:400); anti-MHCII (rat monoclonal, M5/114.15.2, 14-5321-82, eBioscience, 1:400); anti-B220 (rat monoclonal, RA3-6B2, 553084, BD, 1:400); anti-PLVAP (rat monoclonal, MECA-32, 550563, BD, 1:400); anti-CCL21 (goat polyclonal, AF457, R&D, 1:400); anti-PDGFRβ (rat monoclonal, APB5, ab91066, Abcam, 1:400); anti-vWF (rabbit polyclonal, A0082, DAKO, 1:400); anti-VCAM1 (rat monoclonal, 429(MVCAM.A), 550547, BD, 1:400); anti-ICAM1 (rat monoclonal, YN1/1.7.4, ab119871, Abcam, 1:400); anti-Tie2 (goat polyclonal, AF762, R&D, 1:400); anti-podoplanin (mouse monoclonal, D2-40, M3619, DAKO, 1:400); anti-Prox1 (rabbit polyclonal, 102-PA32AG, ReliaTech, 1:400); anti-CGRP (goat polyclonal, ab36001, Abcam, 1:400); anti-TH (rabbit polyclonal, AB152, Millipore, 1:400); anti-claudin5 (rabbit polyclonal, 34-1600, Invitrogen, 1:400); anti-ZO1 (rabbit polyclonal, 61-7300, Invitrogen, 1:400); and Alexa Fluor 488-, 594-, or 647-conjugated anti-rabbit (711-545-152, 711-585-152, 711-605-152), anti-rat (712-545-153, 712-585-153, 712-605-153), anti-mouse (715-545-151, 715-585-151, 715-605-151), anti-goat (705-545-147, 705-585-147, 705-605-147), anti-sheep (713-545-147, 713-585-147, 713-605-147), anti-hamster (127-545-160, 127-585-160, 127-605-160) secondary antibodies (diluted at a ratio of 1:1000) were purchased from Jackson ImmunoResearch.
For visualizing the blood vessel continuity, 25-30 μg fluorescence-labeled antibodies or primary antibodies were used: anti-PODXL (goat polyclonal, AF1556, R&D); anti-PODXL (rat monoclonal, 192703, MAB1556, R&D); anti-Ly6c (rat monoclonal, Monts1, BE0203, BioXcell); anti-endomucin (goat polyclonal, AF4666, R&D); anti-PLVAP (rat monoclonal, MECA-32, 550563, BD); BV421 conjugated anti-CD31 (rat monoclonal, MEC 13.3, 562939, BD). For fluorescence labeling of the antibody, DyLight-Conjugation Kit (abcam) was used according to the manufacturer's instructions.

Validation

All the antibodies were validated for the species (mouse or rat, human) and applications (immunohistochemistry) by the correspondent manufacturer, which is described in the manufacturer's website. Our usage was described in the Methods section of the manuscript as below.
For immunofluorescence staining (IFS), the tissues were permeabilized and blocked with blocking buffer containing 5% donkey serum in 1% Triton-X 100 in PBS for 1 h at room temperature (RT). Then, they were incubated with a primary antibody diluted in the blocking buffer overnight at 4°C. After several washes with PBS, they were incubated with secondary antibodies (Jackson ImmunoResearch) diluted in the blocking buffer for 4 h at RT. After several washes with PBS, they were mounted with Vecta-shield (Vector Laboratories).

Eukaryotic cell lines

Policy information about cell lines and Sex and Gender in Research

Cell line source(s)

African green monkey kidney epithelial cell line (Vero E6, CRL-1586, American Type Cell Culture).

Authentication

We did not do in-house authentication by ourself.

Mycoplasma contamination

Mycoplasma contamination was negative by monthly monitoring with MycoAlert Detection Kit (Lonza).

Commonly misidentified lines
(See ICLAC register)

Not applicable.

Animals and other research organisms

Policy information about studies involving animals; ARRIVE guidelines recommended for reporting animal research, and Sex and Gender in Research

Laboratory animals

Prox1-GFP mouse (Choi, I. et al., Blood, 2011) were provided by Dr. Young-Kwon Hong (Keck School of Medicine of USC) and kept in a C57BL6 and ICR background respectively; Prox1-Cre-ERT2 mice (Bazigou, E. et al., JCI, 2011) were provided by Dr. Taija Mäkinen (Uppsala University) and kept in a C57BL6/J background; VE-cadherin-Cre-ERT2 mice (Okabe, K. et al., Cell, 2014) were provided by Dr. Yoshiaki Kubota (Keio University) and kept in a C57BL6/J; R26-tdTomato (C57BL6/J background) mice were purchased from the

	Jackson Laboratory. Mice were bred and housed in our SPF animal facility with 12 h light/dark cycles, controlled temperature (20-22° C) and humidity (40-60%). Mice were fed a standard chow diet (PMI LabDiet) with water. The ages from embryo day 12.5 to adult and 20-25 months-old aged of the mice were used.<br>Male Syrian hamsters (Mesocricetus auratus) at 6-week-old were obtained from Central Lab Animal (Seoul, South Korea). The hamsters were maintained in an animal BSL3 conditions under optimal physical environments (24 ± 2 °C, 50 ± 5% humidity, 12 h dark/light cycles), and were fed with a standard chow diet (PMI LabDiet).
Wild animals	The study did not involve wild animals.
Reporting on sex	An equal number of both gender mice were used.<br>Exceptionally, only female mice were used in ovalbumin-induced allergic rhinitis mouse model. Becasue, female mice are more susceptible to the development of allergic inflammation than male mice (Melgert, B.N. et al., Clin Exp Allergy, 2005). In addition, only male hamsters were used for generation of the COVID-19 model because the vendor sold only male hamsters for their commercial reason.
Field-collected samples	The study did not invovle samples collected from the field.
Ethics oversight	All animal care and experimental procedures were complied with all ethical regulations for animal research and testing under the approvals by the Institutional Animal Care and Use Committee (No. KA2021-045) of Korea Advanced Institute of Science and Technology (KAIST) and by the Animal Ethics Committee of Jeonbuk National University (Approval No. JBNU-2020-133).

Note that full information on the approval of the study protocol must also be provided in the manuscript.
